# A New Species of *Nyanzachoerus* (Cetartiodactyla: Suidae) from the Late Miocene Toros-Ménalla, Chad, Central Africa

**DOI:** 10.1371/journal.pone.0103221

**Published:** 2014-08-27

**Authors:** Jean-Renaud Boisserie, Antoine Souron, Hassane Taïsso Mackaye, Andossa Likius, Patrick Vignaud, Michel Brunet

**Affiliations:** 1 Institut de paléoprimatologie, Paléontologie Humaine: Évolution et Paléoenvironnements UMR 7262, CNRS and Université de Poitiers, Poitiers, France; 2 Human Evolution Research Center, University of California, Berkeley, California, United States of America; 3 Département de Paléontologie, Université de N'Djamena, N'Djamena, Chad; 4 Chaire de Paléontologie Humaine, Collège de France, Paris, France; University of Florence, Italy

## Abstract

During the latest Miocene and the early Pliocene, tetraconodontine suids were the most predominant large omnivorous mammals in Africa. Yet, new species were often identified on the grounds of limited evidence, a situation impacting their value for biochronological correlations as well as for environmental and biogeographical reconstructions. The description of the most abundant known collection of craniodental remains attributed to the tetraconodontine *Nyanzachoerus* helps to improve this situation. These specimens were collected in the upper Miocene deposits at Toros-Ménalla, northern Chad, central Africa, by the Mission Paléoanthropologique Franco-Tchadienne. We compared them with *Nyanzachoerus* from eastern and southern Africa, using extant species as a reference for patterns of morphological variation. Thanks to a large sample of observations, our work focused as much on craniomandibular morphology as on dental morphology and metrics (improved by an index scoring for the complexity of distal third molars and a detailed investigation of premolar-molar ratios). We recognized two taxa at Toros-Ménalla: *Nyanzachoerus khinzir* nov. sp. and *Ny.* cf. *australis*. We also revised the taxonomic status for other species, including: the restriction of *Ny. syrticus* to its holotype specimen from Sahabi (Libya), the resurrection of the nomen *Ny. tulotos*, and the synonymy of *Ny. kuseralensis* with *Ny. waylandi*. At Toros-Ménalla, *Ny. khinzir* was the only suid coexisting with the anthracotheriid *Libycosaurus* and the hominid *Sahelanthropus*, whereas *Ny.* cf. *australis* was associated with a different, probably younger faunal context. *Nyanzachoerus. khinzir*, which probably had a diversified diet, supports a latest Miocene biogeographical distinction between central Africa and eastern Africa.

## Introduction

The Suidae have played an important role in building biochronological frames for late Neogene African sites [1]–[5]. Nevertheless, African suid species have often been defined on the basis of limited evidence, and with a particular reliance on third molar morphology displaying important intraspecific variations. The tetraconodont genus *Nyanzachoerus* Leakey, 1958 [6] provides a good illustration of this. To echo the caveats of White [7], out of the 11 species most commonly cited in the literature since 1958, seven (*Ny. syrticus*, *Ny. kanamensis*, *Ny. devauxi*, *Ny. waylandi*, *Ny. jaegeri*, *Ny. cookei*, *Ny. kuseralensis*) were initially defined on the grounds of very few dentognathic specimens, most often from a single individual and/or from an imprecise stratigraphic context [6], [8]–[13]. Three of the four remaining species (*Ny. tulotos*, *Ny. plicatus*, and *Ny. pattersoni*) initially based on abundant material [14] are now most often considered synonymous with species from the first group, whereas the last one, *Ny. australis*, is considered by some authors as a subspecies of *Ny. kanamensis*, including in its initial description [1], [15], [16].

It is thus not surprising that relatively recent contributions propose significantly different appraisals of the diversity and relationships within *Nyanzachoerus* [1], [10], [16]–[18], and that, to date, no formal, cladistic phylogenetic analysis of African tetraconodonts has been published. Although these large and abundant omnivores possibly occupied a significant niche in African Mio-Pliocene ecosystems, this current lack of systematic and phylogenetic consensus impedes their use in paleoenvironmental reconstructions, as well as casts doubts on their merits for building biochronological and paleobiogeographical hypotheses.

In this work we propose to erect yet another species of *Nyanzachoerus*, based on fossils discovered at Toros-Ménalla in the Djourab desert, northern Chad, by the Mission Paléoanthropologique Franco-Tchadienne. The remains of this species are particularly abundant, including several partial crania and mandibles, as well as numerous dental rows. The description of this material was an opportunity for assessing morphological patterns of variation within a well-documented species of *Nyanzachoerus*, an important step in assessing the diversity of *Nyanzachoerus* during the latest Miocene and the early Pliocene.

### Geological and paleontological setting

Toros-Ménalla is a more or less flat area located in the northern Lake Chad basin, 500 km north-northeast of Ndjamena, just above the 16^th^ northern parallel and between the 17^th^ and the 18^th^ eastern meridians. The Toros-Ménalla fossiliferous area, abbreviated hereafter “TM”, comprises outcrops separated and periodically overlain by shifting aeolian sand blankets and dunes [19]. Separate outcrops are generally identified as different localities, unless correlations were established between them, a particularly difficult task given the locally level topography. About 400 localities have thus far been designated at TM. Most of these share a particular vertebrate association including a relatively primitive *Nyanzachoerus*, initially attributed to *Ny. syrticus* by Vignaud et al. [19]; the anthracotheriid *Libycosaurus* [19]–[21]; the proboscideans *Anancus kenyensis* and *Loxodonta cookei* Sanders, 2007 [22], both reported by Vignaud et al. ([19]: 154), the latter as *Loxodonta* sp. aff. *L*. sp. indet. ‘Lukeino stage’; the hippopotamid *Hexaprotodon garyam* described by Boisserie et al. [23]; the Hippotragini *Tchadotragus* and *Saheloryx* named by Geraads et al. [24]; as well as *Sahelanthropus tchadensis*, currently the earliest known hominid [25], [26]. Initially, Vignaud et al. [19] biochronogically estimated this faunal assemblage to be older than that of the Lukeino Formation (Baringo Basin, Kenya), i.e. older than 6.0 Ma [27], and correlated it with that of the Lower Member of the Nawata Formation at Lothagam, dated between 7.4 Ma and 6.5 Ma [28]. Subsequently, radiochronological ages were proposed for the hominid localities of TM between 7.2 Ma and 6.8 Ma [29] and between 7.5 Ma and 7.1 Ma [30].

## Materials and Methods

### Material

The collection of craniodental remains of *Nyanzachoerus* from TM is the largest recorded from a single research area. Among 417 collected craniodental specimens (Text S1, Table S7), 294 were well-enough preserved for being identified at a specific level – including 171 measured third molars. For comparison, the next largest published collection of *Nyanzachoerus* is from Lothagam, with 200 listed specimens, included 74 measured third molars deriving from a more species-diverse assemblage and a longer chronostratigraphic interval [17].

State of preservation at TM is extremely variable, notably because of erosive sand-bearing winds that quickly abrade specimens once they are exposed to the surface. Incisors and canines of *Nyanzachoerus* from TM were systematically weathered, broken, and/or found isolated; we do not consider them here for systematic assessments. Suid postcranial elements were also found at TM, but never in direct association with craniodental remains. They were left aside in the present work, and will be dealt with in subsequent studies.

This study was performed within the framework of a research partnership between the University of N'Djamena (Chad), the Centre National d'Appui à la Recherche in N'Djamena (Chad), and the University of Poitiers (France). All necessary permits were obtained for the described study, which complied with all relevant regulations. The final repository of all specimens collected at TM (from 1997 until the present contribution) is the Centre National d'Appui à la Recherche in N'Djamena, Chad.

#### Fossil reference

Direct comparisons were mainly performed with relevant material from five of the most prominent nyanzachoere assemblages from the late Miocene–early Pliocene of Africa: the Nawata Formation at Lothagam, Turkana Basin, Kenya, dated between 7.4 Ma and ca. 5 Ma [14], [17]; the Adu-Asa Formation and the Sagantole Formation (Kuseralee Member) in the Middle Awash Valley, Ethiopia, dated to ca. 5.8 Ma and ca. 5.2 Ma, respectively [10]; the late Miocene-lower Pliocene Sahabi, Libya [9], [31], [32], [33]; Langebaanweg, South Africa, dated to ca. 5 Ma [15]; Kanapoi, dated between 4.17 Ma and 4.07 Ma [14], [34].

These collections include material from seven species of *Nyanzachoerus* with which the TM material was compared in detail. These are: *Ny. syrticus* (Leonardi, 1952) [13]; *Ny. kanamensis* Leakey, 1958 [6]; *Ny. devauxi* (Arambourg, 1968) [11]; *Ny. tulotos* Cooke and Ewer, 1972 [14]; *Ny. waylandi* (Cooke and Coryndon, 1970) [12]; *Ny. australis* Cooke and Hendey, 1992 [15]; *Ny. kuseralensis* Haile-Selassie, 2009 [10]. Specimens from these collections attributed to the above mentioned taxa were directly observed and measured by JRB, using the same protocols as for the Chadian material. Additional morphological and morphometrical comparisons with these and other species were based on published descriptions and metric data [6], [11], [12], [35]–[43].

The designation of these species essentially followed the classification of Tetraconodontinae proposed by Haile-Selassie [10]. Regarding the distinction between *Nyanzachoerus kanamensis* Leakey, 1958 and *Ny. pattersoni* Cooke and Ewer, 1972 [14], we followed the opinion of Harris and White [4], then Cooke [31], Haile-Selassie [10], and the more recent remark of Geraads et al. [44] contra Made [16], Harris and Leakey [17], and Pickford [45], in identifying *Ny. pattersoni* as a junior synonym of *Ny. kanamensis*. Hence the Kanapoi material initially attributed to *Ny. pattersoni* by Cooke and Ewer [14] (see also [46]) is discussed below as *Ny. kanamensis*. We also followed Cooke [31] and Gallai et al. [33] in identifying *Ny. cookei* Kotsakis and Ingino, 1979 [9] as a junior synonym of *Ny. kanamensis*. As explained below, we followed Cooke and Ewer [14], Cooke and Wilkinson [2], and Kotsakis and Inguino [9] in using the name *Ny. tulotos* Cooke and Ewer, 1972 [14] for eastern African material more recently designated as *Ny. syrticus* (e.g., see [1], [10], [16], [17], [19], [31]). In our opinion the name *Ny. syrticus* should be restricted to the holotype specimen. Finally, given the difficulties in applying the concept of subspecies to these fossil assemblages, we avoided using subspecific nomina for fossil taxa (but see: [1], [16]).

#### Extant reference

A large sample of *Potamochoerus* (132 individuals) was used as an extant species reference for intraspecific variation in a contemporary suid species. Sampled specimens are located in the Field Museum of Natural History, Chicago (USA); Forschungsinstitut und Naturmuseum Senckenberg, Frankfurt (Germany); Muséum National d'Histoire Naturelle, Paris (France); Musée Royal de l'Afrique Centrale, Tervuren (Belgium); Museum für Naturkunde, Berlin (Germany); Natural History Museum, London (UK); National Museums of Kenya, Nairobi (Kenya); Naturhistorisches Museum, Bern (Switzerland); South African Museum, Cape Town (South Africa). These data were collected by AS and JRB.

### Methods

Observations reported below are based on the complete suid collection from TM. They describe the major morphological variations observed. Male and female morphotypes were recognized based on the assembly of characters recorded from published data and observations of extant material. These characters include, most prominently: canine size, cranio-mandibular proportions (widening of mandibular symphysis, rostrum, and parietals), and development of cranial superstructures (supracanine flange, cranial roof swellings, zygomatic expansion). For comparisons with other fossil species, we used parameters of third molar size and morphology, ratios within dental rows, and craniomandibular features. Comparative observations reported below mention the most significant specimens as references, but they as well apply to all other specimens observed for the relevant taxa, unless indicated otherwise. We used the variation observed in morphologically comparable extant species of African suids as a referential for descriptions, comparisons, and interpretations of the fossil material. The nomenclature used to describe the teeth is the one proposed by Boisserie et al. [47]: we provided a detailed summary of this nomenclature in Text S2, as well as illustrations in Figure S1.

#### Dental metrics

Teeth were measured according to the protocol defined by Harris and White ([4]: appendix IV). We considered measurements of tooth germs with caution in our interpretations, because they may differ substantially from fully grown teeth. Third molar crown heights were measured for each pair of cusps/-ids as well as for the talon/-id. Heights were taken for the paracone, the metacone, the metaconid, the entoconid, and the highest cusp/-id distal to the metacone-metaconule/hypoconid-entoconid pair, under the strict condition that the measured cusps/-ids had to be unworn to be measured. Given general evolutionary trends observed in African suids and in particular in *Nyanzachoerus* (see, e.g., [4]), ratios involving measurements of isolated teeth, and partial or complete dental rows (notably premolar/molar ratios) were used for exploring the relative proportions of postcanine teeth.

#### Third molar complexity scoring

Third molars are abundant in African tetraconodont collections, and display a strong evolutionary trend of talon/-id elongation through time via the addition of new cusps/-ids (see, e.g., [4]). Third molar morphological features have been therefore used prominently for systematic purposes. However, this marked evolutionary pattern is accompanied by important intraspecific variation. For this reason, we looked for a way to ease comparisons between tooth assemblages. On the basis of our observations of fossil and extant samples, we defined a scoring scale reflecting the complexity of third molars by modifying the model elaborated by Souron [48], the “Complexity Score,” abbreviated hereafter “CS”. For each tooth, the CS was based on a count of main cusps/-ids distal to the metacone-metaconule pair and to the hypoconid-entoconid pair. The CS used in this work for M3 and m3 are defined in Text S3 (see also Fig. S2). We note that CS values do not necessarily reflect tooth size (i.e. large teeth can display a low CS).

#### Paleoecological information

In order to supplement our assessment of tetraconodont diversity at TM, stable carbon and oxygen isotope analyses were used as paleodietary and paleoenvironmental proxies. We here report the analyses on bioapatite carbonate from dental enamel of TM *Nyanzachoerus* conducted by Jacques [49], following the methodology described by Jacques et al. [50]. Twenty-eight individuals of *Nyanzachoerus* were sampled from localities TM 266 and TM 267. These correlated, neighboring localities were initially selected because they represent the richest overall fossil assemblage [19]. Values of δ^13^C and δ^18^O were compared to the previous isotopic results obtained for extant and fossil African suids by Harris and Cerling [51].

#### Nomenclatural acts

The electronic edition of this article conforms to the requirements of the amended International Code of Zoological Nomenclature, and hence the new names contained herein are available under that Code from the electronic edition of this article. This published work and the nomenclatural acts it contains have been registered in ZooBank, the online registration system for the ICZN. The ZooBank LSIDs (Life Science Identifiers) can be resolved and the associated information viewed through any standard web browser by appending the LSID to the prefix “http://zoobank.org/”. The LSID for this publication is: urn:lsid:zoobank.org:pub:A1DAEBF4-CDE3-4AA6-AC0C-68A72D8EAE8F. The electronic edition of this work was published in a journal with an ISSN, and has been archived and is available from the following digital repositories: PubMed Central, LOCKSS.

## Results

### Systematic Paleontology

Cetartiodactyla Montgelard, Catzeflis, and Douzery, 1997 [52]

Suidae Gray, 1821 [53]

Tetraconodontinae Lydekker, 1876 [54]

*Nyanzachoerus* Leakey, 1958 [6]

*Nyanzachoerus khinzir* nov. sp. urn:lsid:zoobank.org:act:98137202-AEF7-4133-9B02-3A3BEF8C981A

Figures 1, 2, 3

**Figure 1:**
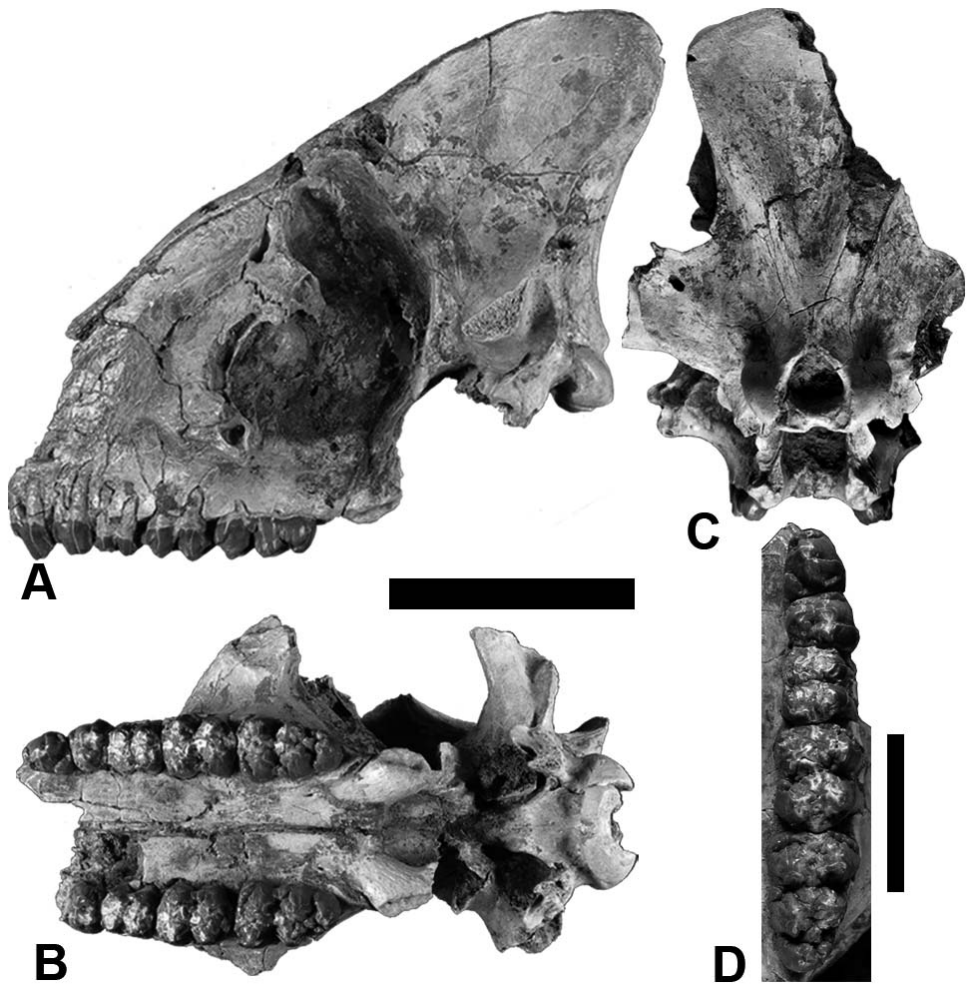
Partial cranium TM 17-97-001, holotype of *Nyanzachoerus khinzir* nov. sp. **A**, lateral view; **B**, ventral view; **C**, nuchal view; **D**, occlusal view of left P3-M3. Scale bars equal 10 cm (**A**–**C**) and 5 cm (**D**).

**Figure 2:**
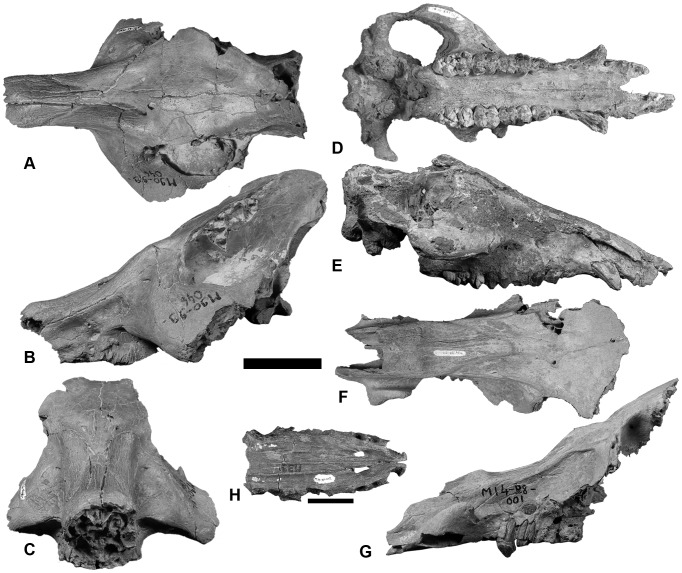
Crania of *Nyanzachoerus khinzir* nov. sp. **A**–**C**, male partial cranium TM 90-99-046 in dorsal (**A**), lateral (**B**), and rostral (**C**) views; **D**–**E**, ventral view of female partial cranium TM 308-01-001 in ventral (**D**) and lateral (**E**) view; **F**–**G**, male partial cranium TM 14-98-001 in dorsal (**F**) and lateral (**G**) views; **H**, rostral portion of palate TM 39-97-002, ventral view. Scale bars equal 10 cm (**A**–**G**) and 5 cm (**H**).

**Figure 3:**
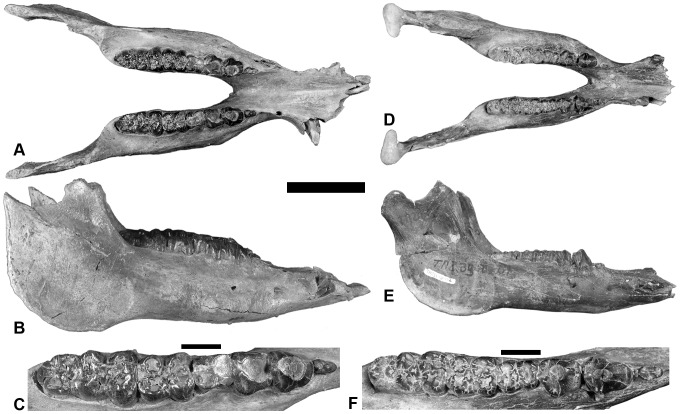
Mandibles of *Nyanzachoerus khinzir* nov. sp. **A**–**C**, male mandible TM 100-00-013 in dorsal (**A**), lateral (**B**), and occlusal view of right p2-m3 (**C**); **D**–**F**, female mandible TM 233-08-001 in dorsal (**D**), lateral (**E**), and occlusal view of right p2-m3 (**F**). Scale bars equal 10 cm (**A**–**B**, **D**–**E**) and 2 cm (**C**, **F**).

“*Nyanzachoerus syrticus*”; Vignaud et al. 2002:154 [19]

#### Holotype

TM 17-97-001, a partial cranium missing the zygomatic arches and the rostral part of the muzzle, bearing the left P3-M3 and the right P4-M3 in moderate to incipient wear (Fig. 1); sex undetermined.

#### Other referred specimens

See specimen list (Text S1), with 279 specimens referred to *Ny. khinzir*.

#### Etymology

“Khinzir” is a transcription of the Chadian Arabic word for “pig”.

#### Occurrence

The species is known from the Toros-Ménalla fossiliferous area, Chad, and more particularly the latest Miocene deposits known as the “anthracotheriid unit” ([19]:153).

#### Diagnosis

A species of *Nyanzachoerus* larger than *Ny. devauxi* and *Ny. waylandi*, smaller than *Ny. australis*, *Ny. kanamensis*, and *Ny. jaegeri*, similar in size to *Ny. tulotos*. Palate absolutely wider than in *Ny. tulotos* and *Ny. devauxi*. In males above mesial premolars, strong dorsal thickening of the nasals forming a continuous swelling, instead of two lateral and/or smaller swellings in other species; moderately enlarged preorbital swellings, unlike in male *Ny. tulotos*. Cranial roof slightly concave to flat instead of markedly concave as in *Ny. tulotos* and *Ny. kanamensis*. Occipital plate and nuchal crest more vertical than in *Ny. tulotos* and *Ny. kanamensis*. Female mandibular corpora shallower than in males, unlike other documented species of *Nyanzachoerus*. Rostral edge of male symphyses not as triangular in dorsal view as in *Ny. tulotos*, but less rounded than in *Ny. australis* and *Ny. kanamensis*. Mandibular symphysis notably less flat than in *Ny. waylandi*. Occurrence of first premolars variable, especially the P1 uni- or bilaterally present in half of the specimens, instead of being always present (*Ny. tulotos*, *Ny. devauxi*), rare (*Ny. australis*), or always absent (*Ny. kanamensis*, *Ny. jaegeri*). When present, first premolars smaller than in *Ny. syrticus*, *Ny. tulotos*, and *Ny. devauxi*, but not as reduced as in *Ny. australis*. On average, upper and lower third molars absolutely larger than in *Ny. devauxi*; smaller, proportionally shorter and lower-crowned than in *Ny. australis*, *Ny. kanamensis*, and *Ny. jaegeri*; more complex than in *Ny. devauxi* and *Ny. tulotos*, less than in *Ny. kanamensis* and *Ny. jaegeri*. Lower third molars less complex than in *Ny. australis*, and on average wider than in *Ny. waylandi*. Premolars larger than in *Ny. waylandi*. Compared to M3, upper premolars relatively narrower than in *Ny. tulotos* and *Ny. devauxi*, relatively larger than in *Ny. kanamensis*, *Ny. australis*, and *Ny. jaegeri*; compared to m3, lower premolars relatively shorter than in *Ny. tulotos* and *Ny. devauxi*, relatively longer than in *Ny. australis* and *Ny. jaegeri*.

#### Taxonomic remarks

The material from TM was initially identified as *Nyanzachoerus syrticus* on the basis of similarities between an initially small sample of Chadian third molars and equivalent dental material from Lothagam [19]. The material from Lothagam was first described under the name *Ny. tulotos* Cooke and Ewer, 1972 [14] as a species differing from *Ny. syrticus* (Leonardi, 1952) [13] from Sahabi (see also [9]). Later, *Ny. tulotos* was synonymized to *Ny. syrticus* by Cooke [31], an opinion shared by most subsequent authors.

We directly re-examined the holotype mandible V. 2004 of *Ny. syrticus* from Sahabi. As described by Kotsakis and Ingino [9], this specimen displays a marked pathological condition: the ventral side of the rostral left corpus presents at least three cavities that may have resulted from a predation-induced wound and/or infectious process. Related bone outgrowth massively distorted this corpus and the symphysis, and may impact the right corpus morphology as well (see [32]: pl. V). Therefore, anatomical comparisons cannot be reliably established between this patently pathological mandible and other mandibular specimens of *Nyanzachoerus*. Cooke [31] based his opinion on the cheek tooth dimensions and proportions, but it seems inappropriate to draw such taxonomically profound conclusions from dental measurements of such a small sample, given the variations observed in extant and fossil suids. We further note that Cooke [31] used similar caution in referring more recently collected, fragmentary suid specimens from Sahabi, to *Ny*. cf. *syrticus*.

Furthermore, given the lack of information on its precise provenience and on its stratigraphic context [13], [32], it is currently not possible to attribute a precise age to this holotype specimen. This mandible was initially described together with a cranium of a more advanced species of *Nyanzachoerus* [9], [31], supporting existing doubts on the contemporaneity of fossil specimens anciently and recently collected at Sahabi (e.g., see [55]).

For all these reasons, in our opinion, comparisons between the holotype mandible of *Ny. syrticus* from Sahabi and material displaying an apparent similar dental evolutionary degree from elsewhere in Africa should not be used to support any significant taxonomic, biochronological, and biogeographical conclusions. We consider *Ny. syrticus* as a nomen dubium that should be restricted to the holotype. Specimens previously referred to *Ny. syrticus* in eastern African sites and known with sufficient specimen samples (principally in the Nawata Formation and in the Adu-Asa Formation) are here referred to *Ny. tulotos* Cooke and Ewer, 1972 [14]. Regarding the two specimens attributed by Cooke [31] to *Ny*. cf. *syrticus*, these specimens (a partial, eroded mandible with left and right p4 and right m2; a left M3) are in a fragmentary condition making difficult to propose a clear attribution. Given the lack of stratigraphic association between these specimens and given our proposed restriction of *Ny. syrticus* to its holotype, it seems more cautious to attribute these two specimens to *Nyanzachoerus* sp., until more complete, diagnostic material is found in the same localities. We reported them in our measurement tables as “*Nyanzachoerus* sp.” from Sahabi.”

### Description and comparisons

#### Cranium (Tables 1 and S1, Figs. 1 and 2)

*Nyanzachoerus khinzir* nov. sp. is represented by six prominent cranial specimens including the holotype partial cranium TM 17-97-001. TM 14-98-001 (Fig. 2F–G) preserves most of a male face, except for the premaxillae and the right supracanine flange. The palate is damaged nuchally to the M1 alveoli. The zygomatic arches, parietals, basicranium, and the occipital plate are missing. All teeth are missing, except the eroded and worn P3. TM 90-99-046 (Fig. 2A–C) is an edentulous male cranium broken in front of the P2 alveoli, missing the lateral-most part of the zygomatic arches and parts of the basicranium. TM 266-01-020 is a fragmentary and eroded male rostrum. The left side retains alveoli from the distal I1 to the M1. The left M2 and M3 are partially preserved and in an advanced stage of wear. The right side is more eroded, only displaying the broken canine and the P1 to P4 alveoli. TM 308-01-001 (Fig. 2D–E) is a female cranium somewhat dorsoventrally crushed, displaying a well-preserved ventral side, including the canines and the left and right P3-M3. The M3s are unworn. The dorsal side is eroded and is lacking the braincase roof. The right premaxilla and the left zygomatic arch are also missing. TM 341-05-007 is a well-preserved palate and eroded basicranium – other parts are missing and the sex is indeterminate. Cranial observations below are based on numerous additional, but more fragmentary, specimens (see Text S1).

The premaxillae are poorly preserved in all specimens except TM 39-97-002 (Fig. 2H), a fragmentary female palate preserving incisor and canine alveoli as well as left P1 and right and left P2. In this specimen, the premaxillae exhibit an ogival shape in ventral view, close to what is seen in *Potamochoerus*. Fragmentary male specimens (notably TM 14-98-001 and TM 266-01-020) indicate a more triangular shape linked to a greater width at the canines. This sexual dimorphism is observed in *Ny. tulotos* from Lothagam (KNM-LT 316 and KNM-LT 23771, respectively male and female crania), and a male specimen of *Ny. devauxi* (KNM-LT 22967) displays a corresponding morphology. In *Ny. australis* from Langebaanweg, the dimorphism is less marked, the female palate SAM-PQL 14429 having premaxillae more triangular than TM 39-97-002 and KNM-LT 23771, with closely similar proportions to those of the premaxillae in male cranium SAM-PQL 22188. In *Ny. kanamensis* from Kanapoi, the female cranium KNM-KP 239 also displays relatively robust premaxillae, more triangular than in TM female specimens. However, the difference with males of *Ny. kanamensis*, which have very broad and robust premaxillae, is marked (crania KNM-KP 264 and KNM-KP 30186). In *Ny. kanamensis* from Sahabi, the female cranium V. 2005 (holotype designated for *Ny. cookei* by Kotsakis and Ingino [9]) also displays broad premaxillae, although more ogival than in KNM-KP 239.

In all observable specimens of *Ny. khinzir*, the premaxillae tend to curve ventrally to the palate. This is congruent with the morphology of *Ny. tulotos* at Lothagam (KNM-LT 316 and KNM-LT 23771) and from the Adu-Asa Formation (STD-VP-1/1), as well as with the morphology of *Ny. australis*. In *Ny. kanamensis*, the female KNM-KP 239 displays a stronger ventral projection, whereas males (KNM-KP 264 and KNM-KP 30186) tend to have more horizontal premaxillae.

At TM, incisor alveoli display some variation. TM 266-01-020 has two subequal I2 and I3 alveoli (the I1 alveolus is incomplete) separated by a short diastema (about 0.5 cm). In TM 308-01-001 and TM 39-97-002, the I3 alveoli are smaller relative to the mesial ones, and the I2–I3 diastema is shorter or even absent. The I1 alveoli are relatively elongate in all observed specimens (only females). In *Ny. tulotos*, KNM-LT 316 differs from TM specimens in having incisor alveoli subequal in size, rounded I1 alveoli, and no inter-incisive diastema; but KNM-LT 23771 is similar to TM 39-97-002. In *Ny. devauxi* (KNM-LT 22967), there is a strong size gradient in incisor alveoli, the I3 being quite reduced. In *Ny. australis*, the I3 alveolus is relatively smaller than the I2 alveolus, and the male I1 alveolus (SAM-PQL 22188) is a bit more rounded than in the female (SAM-PQL 14429). In *Ny. kanamensis*, the male cranium KNM-KP 264 displays a size gradient from large I1 to very reduced I3. The I3 are also very reduced in V. 2005.

In *Ny. khinzir*, females display a narrow rostrum whereas males have an enlarged one. This widening incorporates three main features. First, male canines are larger and slightly shifted laterally compared to the cheek teeth rows (TM 14-98-001, TM 266-01-020) instead of being aligned with I3 and M3 as in female TM 308-01-001. Second, male TM 14-98-001 preserves a strong supracanine flange with a developed dorsal lamina having a gently convex rostrodorsal ridge and a subvertical nuchal ridge. Third, male nasals are thickened. Laterally they are wider at the level of the supracanine flange nuchal extremity (more in TM 266-01-020, less in TM 90-99-046, intermediate in TM 14-98-001). Dorsally, they form a distinctive centered swelling. This thickening seems to involve some pneumatisation of the nasals, as seen on the lateral breaks of TM 266-01-020.

TM 308-01-001 is similar to KNM-LT 23771 in lacking these features or displaying them quite incipiently. In male specimens of *Ny. tulotos*, KNM-LT 316 differs from Chadian specimens by a slightly greater lateral shifting of the canines. Its supracanine flange displays a more elevated vertical lamina about 1 cm and thicker, with a steep rostral ridge forming an angle to a well-developed dorsal rugosity extending about 5 cm. In dorsoventral view the supracanine flange joins the maxilla with a transverse ridge at mesial P2 level, instead of an oblique ridge joining at P2–P3 level as in TM 14-98-001 (Fig. 2F) and TM 266-01-020. The thickening of the nasals is less exaggerated, lacking a clear lateral expansion (closer to TM 90-99-046 in this regard), and displaying two lateral swellings instead of the more centred, larger protuberance seen in the Chadian specimens. Male STD-VP-1/1 has a moderate intercanine widening, a supracanine flange similar in shape but smaller than that of TM 14-98-001, and almost no nasal thickening, except for a slight lateral expansion.

In *Ny. devauxi* (KNM-LT 22967), the intercanine space is narrower; the supracanine flange is more similar in shape to TM 14-98-001, but relatively more robust as in KNM-LT 316; and there is little or no nasal thickening. *Nyanzachoerus australis* differs from TM specimens in having a less marked difference in canine lateral shifting between male (SAM-PQL 22188) and female (SAM-PQL 14429) specimens. In the male, the supracanine flange is more similar to TM 14-98-001 than to KNM-LT 316 but its vertical lamina is nuchally concave. The nasal thickening is principally developed laterally, with a maximum width positioned a bit more rostrally than in TM 14-98-001. Male *Ny. kanamensis* do not display either much canine lateral shifting. The supracanine flange of KNM-KP 264 is relatively weaker and shallower than in *Ny. khinzir*, *Ny. tulotos*, and *Ny. australis*. It is a bit more developed in KNM-KP 30186, but relatively small compared to general size of the specimen. The nasal thickening is almost limited to lateral expansion, more marked in KNM-KP 264 than in KNM-KP 30186.

In addition, male TM 14-98-001 displays a rounded and laterally little expanded lateral edge of the canine alveolus, whereas in KNM-LT 316 and KNM-LT 22967, males of *Ny. tulotos* and *Ny. devauxi* respectively, the lateral edges are markedly laterally expanded. Specimens of *Ny. australis* (SAM-PQL 22188) and *Ny. kanamensis* (KNM-KP 264) are more similar to TM 14-98-001. In female SAM-PQL 14429 of *Ny. australis* and KNM-KP 239 of *Ny. kanamensis*, the upper edge of the canine alveolus is moderately extended and bears a slight crest not observed in *Ny. tulotos* and *Ny. khinzir*.

In *Ny. khinzir*, the cheek tooth rows are usually parallel to slightly laterally arched and marked by the presence of P1–P2 diastemata wherever the P1 are preserved. These diastemata are longer in males (TM 14-98-001, TM 266-01-020) than in females (TM 39-97-002, TM 308-01-001). In *Ny. tulotos*, KNM-LT 316 and STD-VP-1/1 have a P3-M3 series slightly convergent mesially. In male *Ny. devauxi* (KNM-LT 22967), the cheek teeth are subparallel and the P1–P2 diastemata are very short. *Nyanzachoerus australis* has slightly arched to convergent cheek tooth rows and P1–P2 diastemata are much longer in females (SAM-PQL 14429) than in males (SAM-PQL 22188). *Nyanzachoerus kanamensis* has subparallel to laterally arched cheek tooth rows and no observable P1–P2 diastemata due to the lack of P1. The palate is absolutely wider in *Ny. khinzir* than in *Ny. tulotos* and *Ny. devauxi* and is more similar in width to those of *Ny. kanamensis* and *Ny. australis* (Tables 1 and S1).

**Table 1:** Cranial measurements (min.-max. in mm; mean; standard deviation; N) in *Nyanzachoerus*.

Taxa		Cr1	Cr2	Cr3	Cr4	Cr5
TM: *Ny. khinzir*	all	45–51; 48.0; 2	34–46; 38.8; 3.77; 14	49–60; 55.2; 3.26; 14	86–105; 94.4; 4.73; 27	127–148; 135.2; 5.54; 22
	males	51–51; 51.0; 1	38–45; 41.8; 2.99; 4	49–60; 55.4; 5.02; 4	94–97; 95.3; 2	135–137, 136.0; 2
	females		35	60	101	142
TM: *Ny*. cf. *khinzir*				60	111	156
TM: *Ny*. cf. *australis*			40		106	154
*Ny. tulotos*	all	40–43; 41.3; 1.53; 3	29–32; 31.0; 1.73; 3	60–68; 63.7; 4.04; 3	89–91; 90.0; 1.00; 3	131–138, 135.3; 3.79; 3
	LN	40–43; 41.5; 2	29–32; 30.5; 2	63–68; 65.5; 2	89–90; 89.5; 2	131–137, 134; 2
	AA	41*	32*	60*	91*	138*
*Ny. syrticus*						
*Ny. australis*	all		40–44; 41.3; 2.34; 3	42–59; 53.1; 6.74; 8	102–110; 107.4; 3.93; 4	141–155, 149.2; 6.00; 4
	LW		40–44; 42.0; 2	54–59; 56.6; 2.30; 5	108–110; 109.3; 1.15; 3	150–155, 152.0; 2.65; 3
	AA		40	42.1–56.6; 47.2; 3	101.7	140.8
*Ny*. kanamensis (Kanapoi)		51	40–45; 42.5; 2	53–67; 60.0; 2	94–101; 97.5; 2	134–158; 146.0; 2
*Ny. devauxi*			31–33; 32.0; 2	56–58; 57.0; 2	80–84; 82.0; 2	121–125; 123.0; 2
*Ny. kuseralensis*					82	119

Abbreviations: LN, Lower Nawata; AA, Adu-Asa; LW, Langebaanweg;

*, male;

Cr1, orbit length; Cr2, palate width between lingual P4; Cr3, P2–P4 length; Cr4, M1–M3 lenght; Cr5, P3-M3 length.

Dorsally, the nasals of the Chadian specimens display preorbital thickenings (more developed in TM 14-98-001 and TM 90-99-046, less in TM 308-01-001). These preorbital swellings are similar to the condition observed in female *Ny. tulotos* (KNM-LT 23771), in *Ny. devauxi*, and in *Ny. australis*. In males of *Ny. tulotos* (KNM-LT 316, STD-VP-1/1), these swellings are much more developed than in *Ny. khinzir*, particularly in height. In *Ny. kanamensis*, KNM-KP 30186 displays moderate preorbital thickenings, but in KNM-KP 264 they may have been as developed as in KNM-LT 316.

In *Ny. khinzir*, the roots of the zygomatic arches are above M1 (TM 90-99-046, TM 308-01-001) or P4 (TM 266-01-020). In TM 90-99-046, they are robust near the maxilla flange, then taper below the orbit to widen again nuchally. Their broken bases indicate that the zygomatic were well expanded and formed zygomatic protuberances as described for other species of *Nyanzachoerus*. In rostral view, these zygomatic arches are obliquely inserted, the jugal projecting ventrally as well as laterally, and the dorsolateral side of the zygomatic being very steep. In TM 308-01-001, the arches present a constant tapering from the maxillary flange toward the squamosal. They are much less projecting laterally and ventrally. These male/female differences are also observed in *Ny. tulotos*. However, male specimens of *Ny. tulotos* do not have jugals projecting as ventrally as in TM 90-99-046, despite KNM-LT 316 having more expanded zygomatic arches. STD-VP-1/1 displays a projection of its zygomatic arches midway to the conditions seen in KNM-LT 316 and KNM-LT 23771. The latter female specimen differs from TM 308-01-001 in displaying somewhat more projected zygomatic arches, inserted higher on the maxilla flange, with a less steep dorsolateral side. In *Ny. australis*, the male zygomatic arch (SAM-PQL 22188) is well developed, projecting as much as in KNM-LT 316. In rostral view, the left zygomatic arch is oblique as in TM 90-99-046, or even more. However, the medial part of this zygomatic arch is largely reconstructed. On the right side, the dorsolateral side of the zygomatic is less steep than in TM 90-99-046 and initially the lateral extension was maybe not as oblique as reconstructed on the left side. The male crania of *Ny. kanamensis* display the most exaggerated condition, with the lateral expansion of the zygomatic arch forming knobby processes strongly protruding in the horizontal plane, thus differing markedly from the condition of TM specimens. The female cranium KNM-KP 239 has also more developed arches than in TM 308-01-001 and KNM-LT 23771. This development is further exaggerated in V. 2005 from Sahabi.

The orbits reach a position above mid-M3 in TM 17-97-001 and TM 308-01-001, and just nuchally to the M3 in TM 90-99-046. The same kind of variation is observed in *Ny. tulotos*, in which the orbits of the Lothagam specimens reach above mid-M3 whereas the Adu-Asa cranium displays a more nuchal placement of the orbit. SAM-PQL 22188 and SAM-PQL 14429 (*Ny. australis*) display orbits placed as in TM 90-99-046. In *Ny. kanamensis*, the orbits are at least as nuchal as, or even more than in *Ny. australis*. In terms of size (Tables 1 and S1), TM 90-99-046 orbits are absolutely and proportionally larger than in *Ny. tulotos*, but similar in size to those of KNM-KP 30186 (*Ny. kanamensis*). Orbit edges display a jugal notch in TM 90-99-046, *Ny. tulotos* at Lothagam, and *Ny. australis* at Langebaanweg. It is however absent or very reduced in *Ny. kanamensis* from Kanapoi. None of the TM specimens display supraorbital processes as thick as in KNM-LT 316 or STD-VP-1/1: they are thinner, as in KNM-LT 23771. At TM, the lateral extension of these processes is unknown, as they are eroded or broken.

In *Ny. khinzir*, the supraorbital crests are slightly projecting above the temporal fossae (TM 17-97-001, TM 90-99-046), and on average converge less than in *Potamochoerus*, but more than in *Hylochoerus*. Between the crests, the cranial roof of TM specimens is slightly concave (TM 90-99-046) to flat (TM 14-98-001, TM 17-97-001). In *Ny. tulotos*, the supraorbital crests are well projected laterally in males (principally in KNM-LT 316 and to a lesser degree in STD-VP-1/1). The parietals of STD-VP-1/1 are moderately constricted as in the Chadian specimens, whereas KNM-LT 23771 displays a much stronger constriction, as in *Potamochoerus*. To the contrary of TM specimens, the cranial roof is markedly concave in all specimens of *Ny. tulotos* (even in female KNM-LT 23771, rostrally to the sagittal crest). In male crania of *Ny. kanamensis*, this area is broadened and concave at least as much as in KNM-LT 316, thus differing clearly from what is seen in Chad.

The lateral profile of TM crania (TM 14-98-001, TM 17-97-001, TM 90-99-046) is relatively steeper and higher than in KNM-LT 23771 and STD-VP-1/1 (KNM-LT 316 is dorsoventrally crushed), except for TM 308-01-001 (Fig. 2). The height difference is particularly well marked in TM 17-97-001, which also has a more vertical, less nuchally projected occipital plate, as well as occipital condyles set higher up relative to the palate. The profile is more concave in TM 14-98-001 and TM 90-99-046 than in STD-VP-1/1, and the nuchal crest and the occipital plate are more vertical in TM specimens (TM 17-97-001, TM 90-99-046, TM 308-01-001) than in *Ny. tulotos* (STD-VP-1/1 and KNM-LT 23771). In *Ny. kanamensis*, the lateral profile of male crania is also somewhat concave, but the crania are more elongate and the profile is relatively not as steep and high as in TM specimens and *Ny. tulotos*.

In occipital view, the exoccipitals of TM 17-97-001, TM 124-01-003 (a partial basicranium preserving the condyles, the exoccipitals, the basioccipital, and the medial glenoid depressions), and TM 308-01-001 are high, short, and lateroventrally rounded. This contrasts strongly with KNM-LT 316, which has shallower and much more laterally expanded exoccipitals. The exoccipitals of STD-VP-1/1 and *Ny. australis* are more similar in shape to those of the TM material, and KNM-LT 23771 displays an intermediate condition, somewhat closer to that of KNM-LT 316. The strong vertical ridge of the occipital plate found above the foramen magnum in STD-VP-1/1 and KNM-LT 23771 is missing in the TM material (TM 17-97-001, TM 90-99-046). In KNM-KP 30186, the exoccipitals are much wider than in *Ny. khinzir*. In KNM-KP 239, they are less laterally expanded than in the male cranium, but still shallower than in the TM specimens. In both specimens, the supraoccipitals are broader, strongly projected nuchally, and the occipital plate is much more concave than in TM specimens.

The basicranial morphology differs between TM 17-97-001 and TM 308-01-001 on the one hand, and TM 124-01-003 and TM 90-99-046 on the other hand, the latter displaying a narrower, less triangular basioccipital and relatively larger condyles in ventral view. All of these specimens share a vaginal process forming a slender crest protruding laterally and rostrally. In KNM-LT 316, this process is more massive, forming a spike projecting ventrally, and the condyles are more laterally elongate than in the TM material. In *Ny. australis* (SAM-PQL 22188), the condyles are more rostronuchally developed than in TM 17-97-001 and the basioccipital forms a relatively shorter triangle. The vaginal process is more similar to the TM condition. SAM-PQL 22188 further differs in presenting a wider retroglenoid space and glenoid cavities positioned relatively more nuchally than in *Ny. khinzir* and *Ny. tulotos*. KNM-KP 239 (*Ny. kanamensis*) displays glenoids more rostrally positioned than in *Ny. khinzir*. These glenoids are also more ventrally concave, and their nuchal edge is more curved.

#### Mandible (Tables 2 and S2, Figs. 3 and 4)

Many mandibular remains were unearthed at TM and attributed to *Nyanzachoerus khinzir*, principally including several dozen isolated corpora and some isolated symphyses (see Text S1). Some specimens also preserve more substantial symphyseal morphology in association with the corpora. Among these, TM 100-00-013 is a partial mandible of a male (Fig. 3A–C). It includes most of the symphysis (the left canine area is broken), both corpora, and the ventral portions of the rami. It bears the left and right i1, parts of the left c1, and the left and right p2-m3. There is a small alveolus for the right p1. Another one is TM 233-08-001, a complete mandible of a female (Fig. 3E–F). Only the incisors, the canines (broken off the roots), and the left p2 are missing. The rostral part of the symphysis is slightly eroded. The corpora bear the left p3-m3 and the right p2-m3. There are no p1 alveoli on this specimen. The rami are complete, including the condyles and the coronoid apophyses.

**Figure 4:**
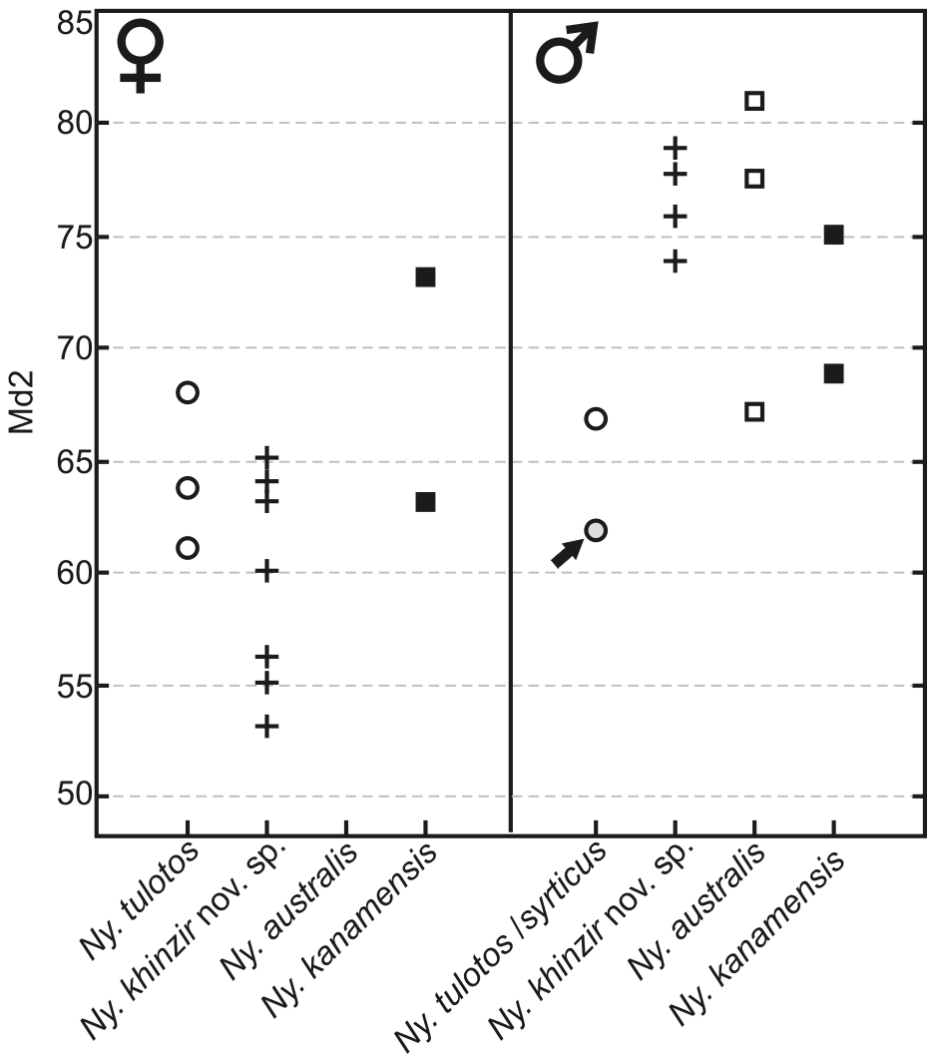
Mandibular height according to sex in species of *Nyanzachoerus*. **Md2**, mandibular corpus height, in mm; **arrow**, holotype of *Ny. syrticus* (V. 2004) from Sahabi.

These specimens illustrate the two mandibular morphotypes recorded for *Ny. khinzir*. According to observations on the extant species, these morphotypes correspond to males and females, respectively. Male mandibles differ from female ones in having a longer and wider symphyseal area, with a less curved dorsal surface. Male corpora are, on average, wider, with an expansion of the corpus lateral side visible in occlusal view, the lateral outline sometimes displaying a net inflexion between the p3 and the m2. In addition, female corpora are markedly shallower than in males, a difference that is not so well expressed in other species of *Nyanzachoerus* (Table 2, Fig. 4).

**Table 2:** Mandibular measurements (min.-max. in mm; mean; standard deviation; N) in *Nyanzachoerus*.

Taxa		Md1	Md2	Md3	Md4	Md5
TM: *Ny. khinzir*	all	247–304; 270.3; 24.1; 4	50–79; 64.3; 7.80; 21	42–56; 48.1; 2.87; 49	84–105; 95.3; 4.86; 43	133–157; 145.1; 6.88; 27
	males	267–304; 285.5; 2	74–79; 76.8; 2.21; 4	46.7–52.3; 49.3; 2.42; 5	88.3–100.6; 95.9; 5.06; 5	134–149.4; 143.2; 6.53; 4
	females	247–263; 255.0; 2	53–65; 59.4; 4.79; 7	46.4–52.8; 48.8; 2.42; 8	92.8–102.6; 96.6; 3.12; 7	143.8–153.3; 146.5; 3.52; 6
TM: *Ny*. cf. *khinzir*		/	/	/	/	/
TM: *Ny*. cf. *australis*		233–278; 255.5; 2	62–70; 66.0; 2	50–54; 52.4; 1.76; 4	97–114; 103.8; 6.78; 5	149–165; 156.5; 7.02; 4
*Ny. tulotos*	all	233	61–77; 66.1; 5.37; 7	47–60; 54.0; 4.00; 12	87–102; 93.0; 5.98; 8	138–156; 145.9; 7.04; 8
	LN		61–68; 63.8; 2.59; 5	47–60; 53.6; 4.20; 10	87–102; 91.7; 5.12; 7	138–156; 144.7; 6.73; 7
	AA		67–77; 72.0; 2	54–58; 56.0; 2	102	154
*Ny. syrticus*		278*	62*	52*	92.7*	149*
*Ny. australis*	all	310	67–81; 74.0; 5.42; 6	45–58; 49.7; 3.53; 15	93–121; 109.2; 8.05; 13	148–172; 159.1; 7.36; 10
	LW	310	67–81; 74.0; 2	47–58; 50.7; 4.32; 6	111–121; 117.1; 4.77; 5	158–172; 165.0; 6.58; 4
	AA		69–78; 74.0; 4.03; 4	44.7–53.3; 49.1; 3.00; 9	93.2–109.3; 104.2; 4.96; 8	147.8–162.2; 155.2; 4.99; 6
*Ny*. kanamensis (Kanapoi)		245–279; 263.4; 13.5; 5	63–75; 69.4; 4.77; 5	45–52; 49.4; 2.94; 7	92–122; 101.6; 9.17; 9	139–165; 148.9; 8.51; 8
*Ny. devauxi*			72	41–44; 42.7; 1.53; 3	75–83; 79.0; 4.00; 3	116–129; 122.5; 9.19; 2
*Ny. waylandi*			57	48	93	139
*Ny. kuseralensis*		217		40	88	123

Abbreviations: LN, Lower Nawata; AA, Adu-Asa; LW, Langebaanweg;

*, male;

Md1, length between rostral extremity of premaxillae and distal m3; Md2, height of corpus at m2-m3; Md3, p3–p4 length; Md4, m1–m3 length; Md5, p3-m3 length.

Canines are also larger in males, but in the case of very old males, size difference may be reduced. For example, TM 186-01-001 associates left and right corpora bearing p3-m3 and p2-m3 respectively, with an eroded symphysis bearing the root of the left lower canine. This specimen is a male, displaying a broad, long symphysis and a strong inflation of the lateral corpora; it is of advanced age, given the advanced wear of its dentition, to the point the m3 labial wall enamel was worn out. The canine transverse section regularly tapers toward its radical extremity, indicating that it probably reached a growth limit with ageing – this is also observed on the size of the right canine alveolus. This canine is of relatively small size.

In most symphyseal specimens, the rostral edge of the incisor process is triangular in dorsoventral view. In females, this triangle is generally more acute than in males. Some specimens (such as TM 79-99-002 and TM 172-01-009) present a more rounded shape, with the incisors closer to being rostrally aligned. In most specimens, incisor alveoli tend to be mesiodistally compressed or bean-shaped for the i1 and the i2, and more circular for the i3. The i2 alveolus is usually the largest. Some specimens display smaller, less compressed and more regularly shaped alveoli for i1 and i2 (e.g., in TM 172-01-009). The canines are obliquely inserted.

The mandible V. 2004 holotype (and sole specimen) of *Ny. syrticus*, belonged most likely to a male and presents a marked pathological condition (see above). The morphology of the right corpus, apparently less impacted by pathological distortion, recalls TM male specimens in its large canines and in being inflated. Yet, this corpus is markedly shallower than in the TM males (Table 2, Fig. 4).

In *Ny. tulotos* from Lothagam, the symphysis is generally triangular (e.g., KNM-LT 287) and no rounded pattern such as in TM 79-99-002 was preserved. It can also be markedly concave ventrally (as in KNM-LT 287, KNM-LT 295, KNM-LT 22996, and V. 2004 from Sahabi) instead of slightly concave to flat in *Ny. khinzir*. The incisor alveoli can be more mesiodistally compressed. The postcanine constriction is also stronger and more nuchally placed in some specimens, but this is not a constant feature. The most prominent difference is the absence of strong sexual dimorphism in corpus height.

All observed specimens of *Ny. australis* from Langebaanweg and the Sagantole Formation display wide symphyses with a rounded rostral edge in dorsal view. Compared to TM 100-00-013, male specimens attributed to *Ny. australis* (SAM-PQL 20490 and SAM-PQL 20552, two complete male mandibles, the latter not being fully adult, and SAM-PQL 21006, a male right hemimandible) display relatively shallower corpora and rostronuchally more developed rami. In Langebaanweg male specimens, the inflation of the corpus is maximal under m2, whereas it is more rostral in TM specimens. AME-VP-1/8 (a partial mandible from the Sagantole Formation) does not display the distinctive tapering of the corpus nuchally to m2 seen at Langebaanweg. Instead, it seems more similar to TM specimens, in reaching more rostrally its maximal inflation, which remains constant nuchally, but the sex of this specimen could not be identified.

*Nyanzachoerus kanamensis* from Kanapoi also retains a wider symphysis with a rounded rostral edge and more aligned incisors, as well as a stronger reduction of the i3. Sexual dimorphism is not extreme, mainly expressed through intercanine widening, but not strongly marked on the corpus as in *Ny. khinzir* (e.g., between male KNM-KP 38978 and female KNM-KP 239). As in *Ny. australis*, the corpus tends to be shallower than in male *Ny. khinzir*.

*Nyanzachoerus waylandi* (partial mandible AMW-VP-1/71) and *Ny. kuseralensis* (partial mandible KUS-VP-1/15) from the Sagantole Formation have shallow corpora, which compares only with female specimens of *Ny. khinzir*. KUS-VP-1/15, the holotype of *Ny. kuseralensis*, has a relatively narrow symphysis with a rounded rostral edge. Ventrally, it differs from *Ny. khinzir* in being regularly convex in continuity with the ventral edge of the corpus, instead of displaying a change of general direction and a more or less pronounced concavity. AMW-VP-1/71 is more fragmentary but presents the same general conformation as the holotype of *Ny. kuseralensis*.

#### Premolars (Tables 3–6, and S3)

The P1 are well preserved in only two specimens of *Nyanzachoerus khinzir*, their presence being otherwise indicated by its remaining double- or single-rooted alveolus. On nine observable specimens, the tooth is unilaterally absent in one occasion (TM 39-97-002), and bilaterally absent in another one (TM 9-01-005). When observable, the P1 is absolutely smaller and proportionally less elongate than in *Ny. devauxi* and *Ny. tulotos* (Tables 3 and S3). Their dimensions are closer to those observed for *Ny. australis*. They are somewhat simpler than in KNM-LT 316, notably displaying less developed cingula. Yet, they are not as bunoid as in *Ny. australis* from Langebaanweg (e.g., on SAM-PQL 22188) and retain a reduced ‘metacone’. These teeth are generally absent in *Ny. kanamensis*.

**Table 3:** P1 and P2 measurements (min.-max. in mm; mean; standard deviation; N) in *Nyanzachoerus*.

Taxa		L P1	w P1	L P2	w P2
TM: *Ny. khinzir*		8.8–9.9; 9.4; 2	5.6–5.7; 5.7; 2	12.4–13.8; 13.1; 0.54; 7	7.2–9.2; 7.8; 0.69; 7
TM: *Ny*. cf. *khinzir*				12.5	8.1
*Ny. tulotos*	all	10.0–11.8; 10.9; 2	5.4–6.4; 5.9; 2	13.0–16.0; 14.5; 1.30; 4	7.4–10.4; 8.9; 1.23; 4
	LN	11.8	6.4	13.9–16; 15.0; 2	9.0–10.4; 9.7; 2
	UN			15.0	8.7
	AA	10.0	5.4	13.0	7.4
*Ny. australis*		9.0–10.4; 9.8; 0.59; 4	5.8–6.5; 6.2; 0.34; 4	10.4–17.4; 13.3; 2.01; 10	5.7–13.4; 8.4; 1.80; 12
*Ny. kanamensis*				9.2–15.0; 12.2; 2.04; 8	6.7–9.9; 8.3; 1.23; 8
*Ny. devauxi*		10.2–10.4; 10.3; 2	4.7–5.8; 5.3; 2	13.7–13.9; 13.8; 2	6.6–8.5; 7.6; 2

Abbreviations: LN, Lower Nawata; UN: Upper Nawata; AA, Adu-Asa; L, mesiodistal length at cervix; w, maximal labiolingual width of crown.

On 20 specimens of *Ny. khinzir* with a preserved c1-p2 area, the presence of p1 was marked by alveoli in 10 cases, out of which two cases indicated unilateral presence only. This pattern of variation differs from what is observed in other nyanzachoere species: p1 alveoli were always present in *Ny. tulotos* (nine specimens) and never in *Ny. kanamensis* (eight specimens); on seven specimens of *Ny. australis*, a single occurrence of the alveoli was recorded. At TM, it can be noted that the p1 are absent in seven specimens identified as females, whereas in six specimens identified as males it is present (three specimens), unilaterally absent (one specimen), or bilaterally absent (two specimens). A single p1 was preserved at TM in TM 246-01-001, a juvenile partial mandible with erupted m2 but fully encrypted m3. This left p1 is simpler and smaller (Table 4) compared to those observed in *Ny. tulotos* from Lothagam and *Ny. syrticus* from Sahabi.

**Table 4:** p1 and p2 measurements (min.-max. in mm; mean; standard deviation; N) in *Nyanzachoerus*.

Taxa		L p1	w p1	L p2	w p2
TM: *Ny*. *khinzir*		8.4	4.9	9.2–16.2; 13.4; 2.03; 11	6.7–9.7; 8.3; 0.98; 12
*Ny*. *tulotos*	all	10.2–14.0; 12.2; 1.91; 3	6.2–6.8; 6.4; 0.32; 3	12.7–19.9; 15.0; 2.29: 9	7.7–8.9; 8.2; 0.39; 9
	LN	12.5–14; 13.3; 2	6.3–6.8; 6.6; 2	12.7–16.4; 14.4; 1.56; 7	7.7–8.6; 8.1; 0.32;7
	UN	10.2	6.2	14.5–19.9; 17.2; 2	7.9–8.9; 8.4; 2
*Ny. syrticus*		11.7	6.8	14.0	9.0
*Ny*. *australis*				10.0–16.6; 13.6; 2.07; 15	7.1–9.5; 8.3; 0.88; 15
*Ny*. *kanamensis*				7.0–15.5; 9.2; 2.10; 15	6.4–9.8; 7.6; 0.86; 15
*Ny*. *devauxi*		10.3	5.1	11.9–15.0; 13.5; 2	5.2–9.0; 7.1; 2
*Ny*. *waylandi*				11.0	5.2

Abbreviations: LN, Lower Nawata; UN: Upper Nawata; L, mesiodistal length at cervix; w, maximal labiolingual width of crown.

The P2 are also somewhat smaller in TM specimens than in *Ny. tulotos*, although they display similar proportions (Tables 3 and S3). The cingula are again less developed, and their occlusal outline is generally more oval as in STD-VP-1/1, instead of triangular as in KNM-LT 316. There are no marked differences with the P2 of *Ny. australis* at Langebaanweg. To the contrary, *Ny. devauxi* and *Ny. kanamensis* are distinct in displaying P2 on average proportionally longer and shorter, respectively (Tables 3 and S3).

The p2 is strongly variable in *Ny. khinzir*. Of 40 observable specimens, the teeth were missing in three specimens. In one adult specimen, TM 170-01-019, no alveoli were found for the p2, but the left corpus, broken obliquely just nuchally to the canine, displayed a p2 totally encrypted, and morphologically distinct from other p2 of *Ny. khinzir* in having a wider distal wall with a strong distal cingulid. In other specimens, the morphology varies from elongate teeth displaying pre- and postprotocristids with a marked distostylid, to bunoid specimens such as in TM 337-02-002. In *Ny. tulotos*, the morphology is similar to that of TM elongate specimens, and on average the teeth are more elongated than in *Ny. khinzir* (Tables 4 and S3). Specimens of *Ny. australis* have on average a simpler morphology, but display proportions similar to those of *Ny. khinzir*. However, the difference is really marked morphologically and metrically only with *Ny. kanamensis*, in which the p2 are simple and mesiodistally very short.

On average values, there are marked differences in the proportions of the P3 between species. *Nyanzachoerus khinzir* has relatively narrow P3 compared to *Ny. tulotos* and *Ny. kanamensis*, displaying closer proportions to the P3 of *Ny. australis* (Table S3). To the contrary, *Ny. devauxi* has more elongated P3. Yet, when observing intraspecific variations, there are some noticeable ones within *Ny. tulotos*. As opposed to the material from Lothagam (mostly from the Lower Member of the Nawata Formation), *Ny. tulotos* from the Adu-Asa Formation displays average values that are similar to those of *Ny. khinzir*.

Morphologically, the TM P3 often display little or no labial cingulum, a relatively large ‘metacone’, and a well-defined ‘protocone’. In *Ny. tulotos* from both Lothagam and the Adu-Asa Formation, the ‘metacone’ is more reduced and the ‘protocone’ is merged into a more developed cingulum. In *Ny. devauxi*, the ‘protocone’ is more distally located and in some cases not distinguishable from a strong distolingual style. This condition can be also observed in *Ny. australis*, but other specimens such as SAM-PQL 22188 are more similar to the condition found in *Ny. tulotos* and *Ny. khinzir*. In *Ny. australis*, the ‘metacone’ is also often well developed. In *Ny. kanamensis*, the tooth tends to be less elongate (Table 5), with a less developed ‘protocone’.

**Table 5:** P3 and P4 measurements (min.-max. in mm; mean; standard deviation; N) in *Nyanzachoerus*.

Taxa		L P3	w P3	L P4	w P4
TM: *Ny. khinzir*		20.4–26.7; 23.2; 1.48; 38	17.2–23.5; 20.1; 1.79; 34	16.0–21.3; 18.5; 1.21; 39	19.6–26.6; 22.9; 1.59; 39
TM: *Ny*. cf. *khinzir*				20.0	25.5
TM: *Ny*. cf. *australis*		27.2	22.2	20.8	26.9
*Ny. tulotos*	all	20.7–26.0; 23.5; 1.66; 12	17.1–25.0; 22.1; 2.37; 12	16.8–23.8; 19.5; 23*	21.0–27.6; 24.4; 22**
	LN	22.1–25.7; 24.2; 1.46; 5	21.8–25.0; 23.6; 1.20; 5	16.8–22.5; 19.6; 1.72; 10	21.0–27.6; 25.2; 2.21; 10
	UN	21.7	20.2	17.1–21.7; 18.8; 2.16; 4	21.7–25.1; 23.4; 1.71; 3
	AA	20.7–26.0; 23.0; 2.20; 4	17.1–21.6; 19.7; 1.93; 4	18.0–20.9; 19.3; 1.05; 5	22.1–25.8; 23.7; 1.35; 5
*Ny. australis*		20.7–28.7; 24.4; 2.07; 27**	18.5–29.4; 21.7; 2.36; 29**	16.8–23.2; 19.9; 1.60; 27***	20.4–30.0; 24.2; 2.49; 27*
*Ny. kanamensis*		20.2–27.8; 22.7; 2.35; 21°	18.2–25.2; 21.4; 2.14; 21*	17.0–21.0; 19.1; 1.04; 22°	21.2–26.3; 23.7; 1.53; 20°
*Ny. devauxi*		21.3–23.4; 22.4; 1.05; 5	17.3–18.9; 18.1; 0.79; 4	17.6–18.7; 18.0; 0.59; 3	20.4–24.1; 22.3; 1.86; 3
*Ny. waylandi*		16.7	13.9	15.6–16.1; 15.9; 2	18.6–19.6; 19.1; 2

Abbreviations: LN, Lower Nawata; UN: Upper Nawata; AA, Adu-Asa; L, mesiodistal length at cervix; w, maximal labiolingual width of crown; difference with sample of *Ny. khinzir* (t-test):

°, non-significant;

*, p<0.05;

**, p<0.01;

***: p<0.001.

The p3 of *Ny. khinzir* are on average absolutely and relatively narrower than in *Ny. tulotos*, displaying values that are closer to those of *Ny. australis* and *Ny. kanamensis* (Tables 6 and S3). The most striking departures from TM specimens are found in *Ny. waylandi* and *Ny. kuseralensis*, displaying absolutely smaller and proportionally narrower p3. Again, variations are observed within *Ny. tulotos*, with specimens from the Adu-Asa Formation having values much closer to those of TM specimens.

**Table 6:** p3 and p4 measurements (min.-max. in mm; mean; standard deviation; N) in *Nyanzachoerus*.

Taxa		L p3	w p3	L p4	w p4
TM: *Ny. khinzir* nov. sp.		22.5–29.5; 24.8; 1.60; 40	16.4–23.1; 19.8; 1.55; 36	17.4–24.9; 21.7; 1.26; 52	18.1–24.7; 20.7; 1.44; 47
TM: *Ny*. cf. *australis*		24.9–27.6; 26.0; 1.44; 3	20.4–21.7; 21.1; 2	22.8–24.0; 23.6; 0.67; 3	22.4–22.6; 22.5; 2
*Ny. tulotos*	all	21.2–30.5; 25.6; 2.27; 23°	17.6–27.3; 21.8; 2.59; 21***	19.7–27.2; 23.1; 1.58; 30***	17.6–25.5; 21.6; 1.86; 28*
	LN	21.3–28; 25.1; 2.12; 10	18.4–24.5; 21.8; 2.10; 10	21.8–27.2; 24.0; 1.32;18	19.6–25.5; 22.4; 1.57; 17
	UN	23.6–30.5; 27.0; 2.07; 8	20.0–27.3; 23.4; 2.48; 7	20.3–21.1; 20.7; 2	23.5
	AA	21.2–23.6; 22.4; 2	17.0	19.7–23.3; 22.4; 1.70; 6	17.6–20.9; 19.6; 1.20; 6
*Ny. syrticus*		24.2	20.4	23.8	21.0
*Nyanzachoerus* sp. from Sahabi				24.9	26.1
*Ny. australis*		22.8–30.9; 26.2; 2.15; 32**	16.4–25.0; 20.3; 2.37; 30°	19–27.6; 22.8; 2.15; 36**	17.1–26.1; 21.3; 2.14; 37°
*Ny. kanamensis*		20.2–29.0; 24.0; 1.81; 33°	15.0–25.0; 19.4; 2.13; 33°	18.0–27.0; 21.5; 1.83; 41°	15.6–25.0; 19.6; 2.04; 42**
*Ny. devauxi*		18.2–25.0; 21.5; 2.31; 6	15.4–18.0; 16.6; 1.13; 5	19.1–22.6; 20.9; 1.13; 10	16.6–21.5; 19.0; 1.52; 8
*Ny. waylandi*		21.0–22.1; 21.6; 2	12.0–15.7; 13.9; 2	18.2–21.3; 20.2; 1.71; 3	14.0–16.9; 15.1; 1.55; 3
*Ny. kuseralensis*		19.9	13.7	18.7	14.2

Abbreviations: UN: Upper Nawata; AA, Adu-Asa; L, mesiodistal length at cervix; w, maximal labiolingual width of crown; LN, Lower Nawata; difference with sample of *Ny. khinzir* (t-test):

° , non-significant;

*, p<0.05;

**, p<0.01;

***: p<0.001.

Unlike the P3, the P4 present little proportional differences between species (Table S3). Their absolute dimensions are on average significantly smaller in *Ny. khinzir* than in *Ny. tulotos* (except for specimens from Adu-Asa and Upper Nawata) and *Ny. australis*, whereas size differences between *Ny. khinzir* and *Ny. kanamensis* are not significant (Table 5). *Nyanzachoerus devauxi* and especially *Ny. waylandi* display smaller dimensions than in *Ny. khinzir*. Morphological differences between species are minor.

The p4 of *Ny. khinzir* are on average absolutely shorter than in *Ny. tulotos* (Tables 6 and S3). Yet again, the same pattern observed for p3 between Lothagam and Adu-Asa specimens is observed for these teeth. The p4 of *Ny. waylandi* and *Ny. kuseralensis* are also readily distinguishable from those of *Ny. khinzir* in their smaller size and relative compression.

#### Molars (Tables 7, 8, S4, and S5; Figs. 5 and 6)

The first and second molars display a significant deal of variation in cingulum/-id extension and style/-id occurrence. Such variations are observed within all compared species of *Nyanzachoerus*, and the morphology of these teeth is therefore not very helpful in departing different species. Regarding measurements (Table S4), the M1 of *Ny. khinzir* are on average shorter than those of *Ny. tulotos* and closer in length to those of *Ny. kanamensis*. The M2 are, in contrast, more comparable with that of *Ny. tulotos* in absolute dimensions and in proportions. Yet, differences are minor and the m1 and m2 have even less separated metric distributions between species. In contrast, the third molars are by far more variable and more informative.

**Figure 5:**
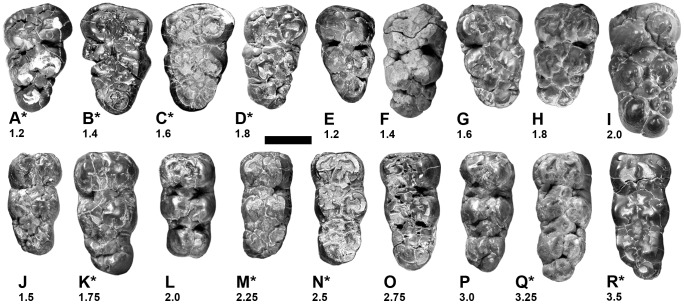
Third molars of *Nyanzachoerus khinzir* nov. sp and *Ny.* cf. *khinzir*. **A–I**, upper molars (*Ny. khinzir*: **A**, TM 92-01-038; **B**, TM 266-03-293; **C**, TM 266-11-028; **D**, TM 92-01-039; **E**, TM 9-01-527; **F**, TM 9-01-528; **G**, TM 9-01-039; **H**, TM 9-01-017; *Ny*. cf. *khinzir*: **I**, TM 9-01-019); **J–R**, lower molars (*Ny. khinzir*: **J**, TM 340-02-001; **K**, TM 254-01-001; **L**, TM 9-01-411; **M**, TM 244-01-002; **N**, TM 171-01-031; **O**, TM 39-99-003; **P**, TM 9-01-021; **Q**, TM 195-01-022; **R**, TM 254-04-003); *, specimens from anthracotheriid-rich sites (others are from anthracotheriid-depleted sites). Complexity scores are indicated for each tooth. Scale bar equals 2 cm.

In *Ny. khinzir*, the simplest M3 (CS of 1.2, Fig. 5A, E) display a talon dominated by a strong distocone accompanied by two or three postectostyles extending labially on at least half of the distocone, occasionally a strong postentostyle, and a small or no median conule. The most complex M3 (CS of 1.8, Fig. 5D, H) present a talon dominated by large postectostyles, extending beyond the labial wall of the distocone to form the crown distal-most extremity. There can be one to several median conules, occupying a significant space. Intermediate scores of complexity are also represented (Fig. 5B–C, F–G), all in equal proportions at TM.

**Figure 6:**
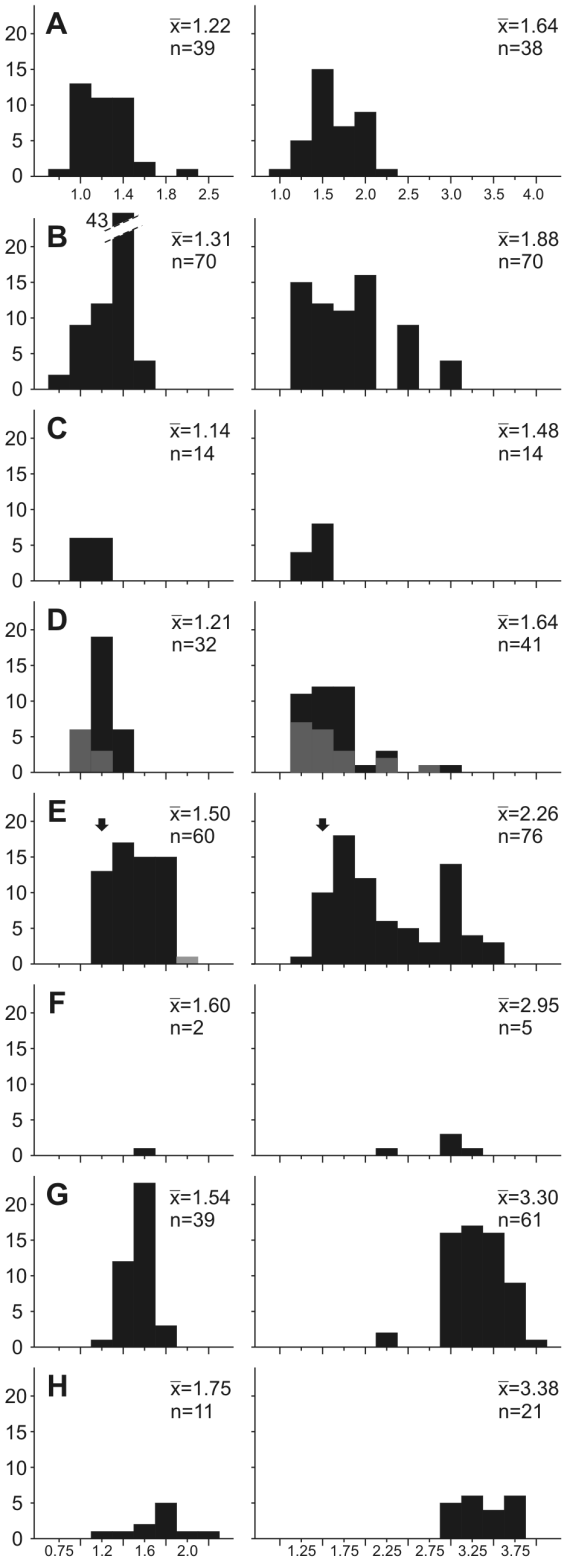
Distribution of third molar complexity scores in species of *Nyanzachoerus*. **A**, *Potamochoerus porcus* (extant comparative data); **B**, *P. larvatus* (extant comparative data); **C**, *Ny. devauxi*; **D**, *Ny. tulotos*, with grey bars for Lower Nawata specimens; **E**, *Ny. khinzir* nov. sp., with grey bar for TM 9-01-019 (*Ny*. cf. *khinzir*) and arrows indicating the position of the single m3 of *Ny. syrticus* and of a single M3 attributed to *Ny*. cf. *syrticus* by Cooke [31]; **F**, *Ny*. cf. *australis* from TM; **G**, *Ny. australis*; **H**, *Ny. kanamensis*. Left column, M3 CS; right column, m3 CS; 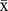, mean; n, specimen number.

**Figure 7:**
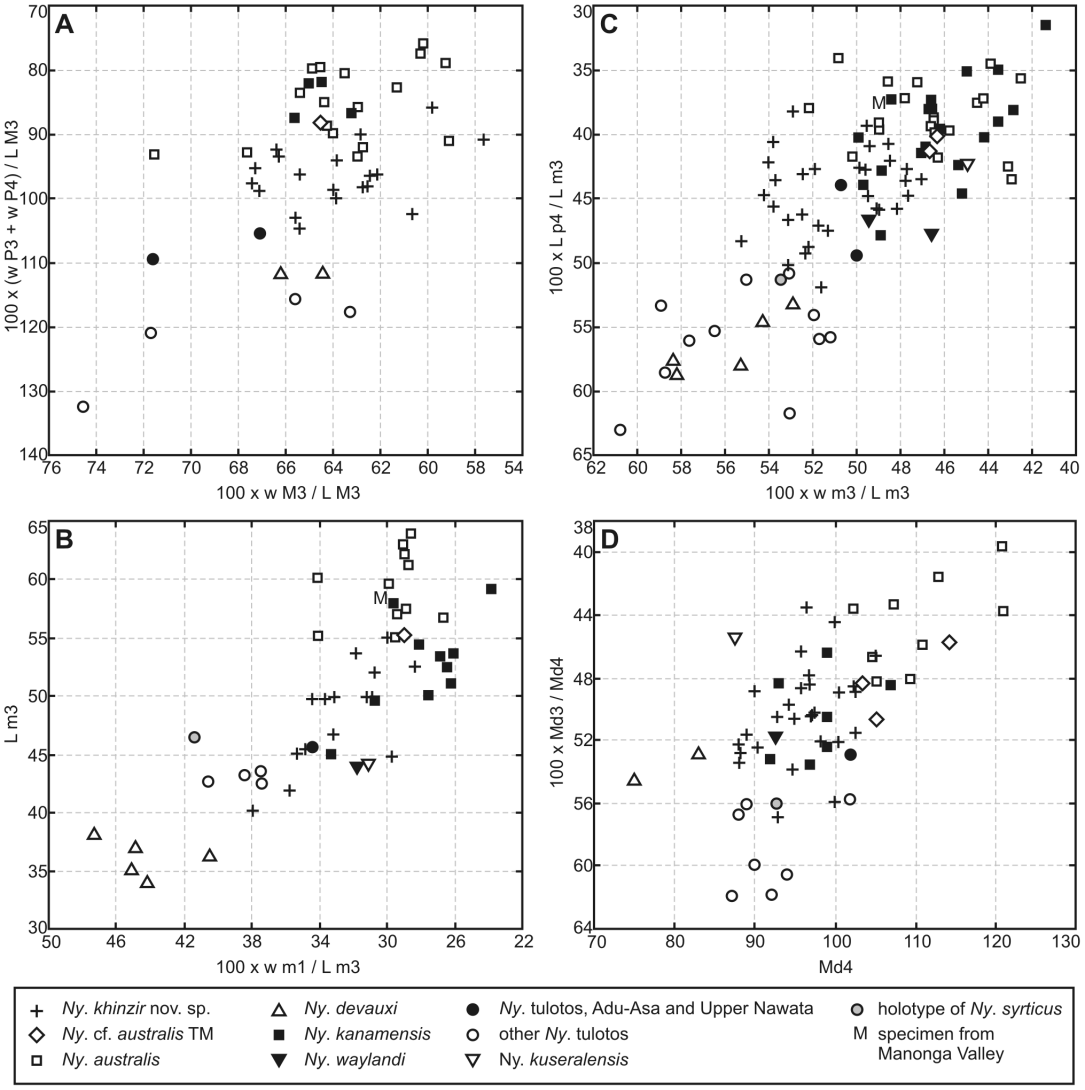
Mandibular and dental proportions in *Nyanzachoerus*. **A**, plot of 100*(width of M3/length of M3) vs. 100*(width of P3 + width of P4)/(length of M3); **B**, plot of 100*(width of m1/length of m3) vs. length of m3 in mm; **C**, plot of 100*(width of m3/length of m3) vs. 100*(length of p4/length of m3); **D**, plot of length of lower molar row (Md4, in mm) vs. 100*(length of lower premolar row/length of lower molar row).

Some specimens of *Ny. tulotos* and *Ny. devauxi* from the Lower Nawata display a simple morphology not recorded in *Ny. khinzir*: a strong distocone accompanied by small styles, or no styles (CS = 1.0). In addition, in *Ny. khinzir*, half of the specimens display a CS of 1.6 or more, whereas such CS are absent in *Ny. devauxi* and only one specimen of *Ny. tulotos* present a CS higher than 1.4 (i.e., 3% of the sample, Fig. 6D). The average score for *Ny. devauxi* and *Ny. tulotos*, all sites included, is 1.1 and 1.2 respectively, whereas it is 1.5 for *Ny. khinzir*. Both species also have mesiodistally shorter M3 on average. The overlap of M3 length between *Ny. khinzir* and *Ny. devauxi* is minimal (Table 7), but it is substantial between *Ny. khinzir* and *Ny. tulotos*. In particular, the latest Miocene samples from the Adu-Asa Formation and the Upper Nawata have distributions more comparable to that of *Ny. khinzir* (Table 7). Yet, the M3s of *Ny. khinzir* are on average more elongate, as shown by the distribution of the width/length index (Fig. 7A), and the occurrence of relatively large and/or multiple median conules is more frequent in *Ny. khinzir* than in *Ny. tulotos*.

**Table 7:** M3 measurements (min.-max. in mm; mean; standard deviation; N) in *Nyanzachoerus*.

Taxa		L M3	w M3	h-tal M3	100*w/L M3
TM: *Ny. khinzir*		39.3–50.9; 44.9; 2.53; 52	23.2–32.9; 28.5; 2.16; 50	14.0–18.6; 16.8; 1.37; 13	57.6–70.6; 63.8; 3.00; 45
TM: *Ny*. cf. *khinzir*		55.6	31.5	20.3	56.7
TM: *Ny*. cf. *australis*		49.5–55.8; 52.6; 3.15; 3	32.7–36.0; 34.1; 1.69; 3	23.2–24.5; 23.9; 2	64.1–66.1; 64.9; 1.05; 3
*Ny. tulotos*	all	37.3–47.6; 42.8; 2.62; 29***	26.4–34.2; 29.5; 1.99; 31*	12.8–19.6; 16.3; 1.98; 18	63.3–77.7; 68.8; 3.63; 24***
	LN	37.3–44.1; 40.5; 2.42; 8	27.1–30.6; 28.2; 1.36; 5	12.8–14.2; 13.5; 2	63.3–77.7; 70.5; 6.02; 5
	UN	39.6–47.6; 44.1; 2.48; 9	27.7–34.2; 31.4; 1.92; 8	14.5–18.8; 16.6; 1.55; 6	67.1–75.2; 69.5; 2.90; 7
	AA	40.7–46.1; 43.0; 1.83; 6	26.4–31.0; 28.9; 1.63; 10	13.2–19.6; 16.9; 2.29; 7	64.9–71.6; 68.1; 2.78; 6
*Nyanzachoerus* sp. from Sahabi		43.7	29.6		67.7
*Ny. australis*		45.1–61.4; 51.3; 4.46; 37***	28.1–39.0; 33.0; 3.10; 39***	17.8–25.1; 20.6; 2.24; 11	59.0–71.6; 63.9; 2.85; 37°
*Ny. kanamensis*		42.7–57.8; 51.8; 3.22; 33***	26.5–42.2; 32.0; 3.49; 26***	19.0–24.2; 21.6; 2	50.8–68.7; 61.1; 4.51; 25**
*Ny. devauxi*		29.5–39.7; 35.3; 3.08; 15	19.7–25.8; 23.6; 1.75; 13	10.6–13.7; 12.4; 1.45; 4	64.4–74.6; 68.3; 2.80; 13
*Ny. waylandi*		31.0–48.0; 38.7; 8.62; 3	22.8–24.5; 23.4; 0.93; 3		51.0–73.5; 62.3; 11.25; 3
*Ny. kuseralensis*		43.5–43.6; 43.6; 2	27.6	14–20.9; 17.5; 2	63.3

Abbreviations: LN, Lower Nawata; UN: Upper Nawata; AA, Adu-Asa; L, mesiodistal length at cervix; w, labiolingual width of mesial lobe of crown; h-tal, maximal height of the talon; difference with sample of *Ny. khinzir* (t-test):

° , non-significant;

*, p<0.05;

**, p<0.01;

***: p<0.001.

The M3 of *Ny. australis* display the same range of complexity as *Ny. khinzir*, with the same mean CS of 1.5 (Fig. 6) and a similar pattern of median conule occurrence. Their proportions are also similar (Fig. 7A), although they are on average absolutely larger than in *Ny. khinzir* (Table 7). On the contrary, the M3 of *Ny. kanamensis* are overall more complex (average CS of 1.8, reaching up to 2.5, Fig. 6) and absolutely and relatively longer (Table 7, Fig. 7A). Both species differ from *Ny. khinzir* in having higher-crowned M3 (Tables 7 and S5).

The m3 of *Ny. khinzir* display a wide range of morphologies, from retaining a large hypoconulid only accompanied by large stylids distally reaching not further than half of the hypoconulid, to talonids including two pairs of cuspids plus a distoconulid and several large stylids. All intermediate degrees of complexity are found at TM (Fig. 5), with a bimodal distribution of CS at 1.75 and 3.0, and a mean of 2.3 (Fig. 6). Median conulids are generally reduced compared to the rest of the talonid, although some specimens can display relatively large and/or multiple ones (Fig. 5N, O).

Again, *Ny. tulotos* and *Ny. devauxi* have quite similar complexities for the m3, with mean CSs of 1.6 and 1.5 respectively, i.e. less than the first mode observed for the CS of *Ny. khinzir*. The CS ranges are also less extended, especially for *Ny. devauxi* (Fig. 6). The absolute dimensions of the m3 of *Ny. khinzir* are slightly higher on average than those of *Ny. tulotos*, and clearly higher than those of *Ny. devauxi* (Table 8). Yet, the m3 of *Ny. khinzir* has more elongate proportions than *Ny. tulotos* from the Lower Nawata and *Ny. devauxi* (Fig. 7B–C).

**Table 8:** m3 measurements (min.-max. in mm; mean; standard deviation; N) in *Nyanzachoerus*.

Taxa		L m3	w m3	h-tal m3	100*w/L m3
TM: *Ny. khinzir*		39.1–55.0; 47.9; 3.61; 81	20.7–29.1; 24.4; 1.79; 72	14.1–19.0; 15.9; 1.47; 17	42.3–59.6; 50.8; 3.11; 68
TM: *Ny*. cf. *australis*		55.0–60.0; 57.2; 2.12; 6	24.6–27.8; 26.0; 1.31; 5	18.3–23.8; 21.1; 2	44.2–46.7; 45.7; 0.97; 5
*Ny. tulotos*	all	38.0–51.1; 44.2; 2.80; 32***	21.1–27.0; 24.1; 1.35; 34°	13.2–18.1; 15.6; 1.58; 9	49.3–60.8; 54.0; 3.12; 30***
	LN	38.0–46.4; 42.7; 1.90; 17	21.1–25.3; 23.8; 1.20; 16	13.2–15.4; 14.5; 1.00; 4	51.2–60.8; 55.3; 3.01; 15
	UN	43.3–51.1; 47.2; 3.27; 4	22.6–25.6; 24.4; 0.99; 6	15.9	50.1–53.6; 51.6; 1.55; 4
	AA	42.7–45.8; 44.8; 1.42; 4	22.0–23.2; 22.7; 0.50; 4	15.5–18.1; 17.2; 1.45; 3	49.3–51.6; 50.6; 1.11; 4
*Ny. syrticus*		46.4	24.8		53.4
*Ny. australis*		48.8–67.0; 57.6; 4.57; 47***	22.2–33.3; 26.9; 2.85; 44***	16.6–24.1; 19.7; 2.18; 22	42.5–52.2; 46.7; 2.41; 41***
*Ny. kanamensis*		45.0–66.4; 55.2; 4.27; 66***	22.0–30.8; 25.0; 1.67; 65°	20.8–21.4; 21.1; 2	37.9–54.5; 45.6; 3.08; 62***
*Ny. devauxi*		31.9–39.6; 36.2; 2.34; 12	18.3–22.5; 20.5; 1.57; 14	12.9–13.3; 13.1; 2	52.9–61.8; 56.4; 2.60; 12
*Ny. waylandi*		40.0–51.1; 44.7; 3.51; 7	18.3–24.1; 20.9; 2.09; 7	16.6	44.0–49.5; 46.6; 1.62; 7
*Ny. kuseralensis*		44.3	19.9	18.7	44.9

Abbreviations: LN, Lower Nawata; UN: Upper Nawata; AA, Adu-Asa; L, mesiodistal length at cervix; w, labiolingual width of mesial lobe of crown; h-tal, maximal height of the talonid; difference with sample of Ny. khinzir (t-test):

° , non-significant;

*, p<0.05;

**, p<0.01;

***: p<0.001.

*Nyanzachoerus australis* and *Ny. kanamensis* display on average more complex m3 than *Ny. khinzir*, with larger distribution ranges (Table 8) and average CSs of 3.3 and 3.4 respectively (Fig. 6). They are also higher-crowned to much higher-crowned respectively, *Ny. khinzir* displaying a crown height close to that observed in *Ny. tulotos* (Tables 8 and S5). Finally, the m3 of *Ny. australis* and *Ny. kanamensis* are on average absolutely and proportionally more elongate than in *Ny. khinzir* (Table 8, Fig. 7C).

#### Dental rows, measurements and ratios (Tables 1 and 2, Fig. 7)

Considering the lengths of P3-M3 and p3-m3 as proxies for general body size, *Nyanzachoerus khinzir* compares well with *Ny. tulotos* in general size; it is larger than *Ny. devauxi*, *Ny. waylandi*, and *Ny. kuseralensis*; and smaller than *Ny. australis* and *Ny. kanamensis* (Tables 1 and 2).

In accordance with observations of isolated tooth measurement trends, the postcanine dentition ratios that displayed the most statistically significant discriminations between species were: for the upper series, the relation between the width of premolars and the length of the M3; for the lower and the upper series, the relation between the width of the first molar and the length of the third molar; for the lower series, the relation between the length of the p4 and the length of the m3. The first ratio (Fig. 7A) showed relatively wide premolars in *Ny. devauxi* and *Ny. tulotos*, relatively narrower premolars in *Ny. australis* and *Ny. kanamensis*, and intermediate proportions for *Ny. khinzir*, overlapping with *Ny. australis*. The second ratio (Fig. 7B) discriminates well most species, *Ny. khinzir* displaying intermediate values between *Ny. kanamensis* and *Ny. australis* on the one hand and *Ny. tulotos* on the other hand. The third ratio (Fig. 7C) has a similar pattern to the upper premolar-molar ratio, except that late specimens of *Ny. tulotos* (Adu-Asa, Upper Nawata) have relatively shorter p4, matching those of *Ny. khinzir*.

Another ratio, the relation between the length of the p3–p4 series and the length of the lower molar series (Fig. 7D), distinguishes relatively short lower premolars in *Ny. devauxi* from longer ones in *Ny. tulotos*, and shows a quite limited overlap between *Ny. khinzir*, *Ny. tulotos*, and *Ny. australis*, as well as no distinction between *Ny. khinzir* and *Ny. kanamensis*.

### Systematic Paleontology

*Nyanzachoerus* cf. *khinzir*

Figure 5I

#### Referred specimen

TM 9-01-019, left maxilla with P2, P4-M3.

#### Occurrence

This specimen was found at locality TM 9, together with other suid specimens attributed to *Nyanzachoerus khinzir*.

### Description

The partial left maxilla TM 9-01-019 displays an M3 slightly more advanced than specimens of *Ny. khinzir* (CS of 2.0, Fig. 5I and Table 7). The distal-most postectostyle is almost as large as the distocone and accompanied by numerous smaller postectostyles. This erupting tooth is also markedly longer than other specimens at TM 9, with a mesiodistal length of 55.6 mm. Including this specimen, the dimensions of *Ny. khinzir* would therefore range from 39.3 mm to 55.6 mm for an average length of 45.1 mm instead of 44.9 mm (cf. Table 7).

### Systematic Paleontology

*Nyanzachoerus* cf. *australis* Cooke and Hendey, 1992 [15]

#### Referred specimens

TM 29-97-002, eroded right corpus with p2-m3; TM 70-99-001, tooth fragment, including fragmentary m3; TM 71-99-001, left corpus with p3-m3; TM 72-01-001, eroded right M1–M2, M3 plus left corpus with p3-m2 and m3 partially encrypted; TM 72-01-002, left hemimandible with eroded m2-m3; TM 73-01-005, right corpus with broken p3-m3; TM 193-01-002, left m3; TM 230-01-001, palate with right P3-M3 and left P4-M3 plus eroded right and left corpora with broken p3-m3 and p4-m3, respectively; TM 230-01-002, juvenile left hemimandible with unerupted p4 and m2; TM 230-01-004, eroded right m2; TM 230-01-005, partial mandible; TM 230-01-006, eroded palate with roots of left and right P3-M3; TM 230-01-008, eroded left corpus with m2-m3; TM 230-01-009, eroded right p4-m3 plus eroded left M3.

#### Occurrence

This material was found in a geographical subgroup of TM localities centered on 16.22°N, 17.16°E and isolated from other TM outcrops, called hereafter ‘south TM’. These localities do not present sedimentological differences with northern outcrops but differ in fossiliferous contents, notably lacking anthracotheriids and including a camelid.

### Description and Comparisons

The most complete specimens are TM 230-01-001, a partial palate with right P3-M3 and left P4-M3 (M3 erupting); and TM 72-01-001, a left corpus with the canine root, the p2 alveolus, and p3-m3 (erupting). Despite these specimens being subadults they display overall dimensions that are similar to those of the largest specimens of *Nyanzachoerus khinzir* (Tables 1, 2, and S2). All third molars are complex (Fig. 6F) and higher-crowned than in *Ny. khinzir* (Tables 7, 8, and S5). Their dental series proportions are similar to those of *Ny. australis* and *Ny. kanamensis* (Fig. 7). On TM 72-01-001, the p1 is absent, and the p2 had two independent roots. The average length p2-p4 is 67.7 mm (range 63.9 mm–71.0 mm, N = 3), i.e. similar to that of *Ny. australis* (mean = 66.5 mm, 60.0 mm–73.8 mm, N = 14) and larger than in *Ny. kanamensis* (mean = 58.3 mm, 56.0 mm–64.0 mm, N = 7).

### Systematic Paleontology

*Nyanzachoerus* sp.

#### Referred specimens

See specimen list (Text S1), with 170 specimens referred to *Nyanzachoerus* sp.

#### Occurrence

This material was found in association to *Nyanzachoerus khinzir* at TM.

#### Remark

These specimens are isolated, weathered (notably by sand wind), and/or fragmentary specimens found in the same localities as *Nyanzachoerus khinzir*. Morphological observations did not depart from *Nyanzachoerus*, but a specific attribution was not possible given the preservation status of these specimens.

## Discussion

### Morphological variations within *Nyanzachoerus khinzir*

The material attributed here to *Nyanzachoerus khinzir* is the largest collection of *Nyanzachoerus* found in a single research area to date. Expectedly, *Ny. khinzir* displays significant morphological variation. Yet its craniomandibular and dental measurement ranges are not noticeably greater than those of other well-represented species of *Nyanzachoerus* (Tables 1–8, S1, S2, and S4), and tend to be normally distributed. In particular, this is the case for third molar lengths.

However, three features seem to be unexpectedly variable in *Ny. khinzir*, their pattern of variation being not observed in other species of *Nyanzachoerus*. First, the distribution of M3 CS is clearly bimodal on a large range (from 1.25 to 3.5). Second, the p1 alveoli are absent and present in close proportions. Third, the mandibular corpora of females are shallower than in males.

These three cases were compared to variations observed in the extant *Potamochoerus*. In the first case, extant *P. larvatus* presents a similarly large range (from 1.25 to 3.25, Fig. 6) as in *Ny. khinzir*, as well as a non-normal, multimodal distribution. In other words, some intermediate CS values were less frequently observed than others. This situation could be linked to the scarcity of some complexity scores in relation to their definition and/or to underlying constraints of cuspid organization. This seems to be the case for m3 CS of 2.25 and 2.75.

*Potamochoerus* is relatively more advanced than early species of *Nyanzachoerus* in terms of premolar reduction, and an overwhelming proportion of individuals do not present any p1 (128 out of 130 specimens). Instead, the P1 is more variably present in *Potamochoerus*. In *P. larvatus*, the P1 are indeed variably present, being uni- or bilaterally missing in 35% of 74 specimens. This situation therefore recalls what is observed in *Ny. khinzir* for the p1.

Although with more overlap than for *Ny. khinzir* (but with a larger sample), extant *Potamochoerus* also displays a difference between females and males in corpus height mesially to m3: for males, it is on average 45.9 mm (min = 36.3, max = 53.4, N = 25, SD = 4.7), whereas for females it is on average 41.5 mm (min = 37.7, max = 44.2, N = 14, SD = 2.4), this difference being significant (t-test and Mann-Whitney U-test with p<0.005). It should be noted that in *Ny. khinzir* this feature does not correlate with the variations in CS or in premolar occurrence but systematically co-occurs with sexual dimorphism. It also does not correspond to noticeable interspecific differences between other species of *Nyanzachoerus*. In addition, male and female morphotypes were found associated within localities (TM 79) and correlated sections (TM 254 and TM 266, see [29]).

Another pattern of distribution was explored. The anthracotheriid *Libycosaurus* is considered as a major marker of the TM faunal assemblage and is absent from other Chadian fossiliferous areas as well as from contemporary eastern African sites [19]. Each TM locality including more than 30 specimens was attributed to one of three locality subsets grounded on the abundance of *Libycosaurus*: lacking (N = 13 localities), depleted (less than 5% of the total number of specimens, N = 15), rich (more than 5%, N = 31). It was not possible to differentiate these locality subsets from each other on the grounds of dental measurements and first premolar occurrences observed in *Ny. khinzir*. The full ranges of third molar CS were found in anthracotheriid depleted and rich localities and sometimes in a single locality such as TM 9 and TM 254 (Fig. 5E–I, K, R). Only m3 with low CS were found in localities lacking *Libycosaurus*, but only six specimens could be recorded in this subset, compared to 22 and 26 for depleted and rich localities, respectively.

Within all localities bearing *Ny. khinzir*, a single specimen was found to really stand out: TM 9-01-019. Displaying an unusually long M3 and a CS of 2.0, this more advanced specimen has also slightly higher molar crowns (Tables 7 and S5). Yet, its proportions and hypsodonty indices do not allow a clear attribution of this specimen to a taxon other than *Ny. khinzir*: it could possibly be an unusually large specimen of this species. It is therefore provisionally attributed to *Ny.* cf. *khinzir*. In conclusion, *Ny. khinzir* displays a pattern of variation comparable to intraspecific variability within extant species, lacking correspondence with particular faunal associations within TM. This further suggests that no phyletic trends are required to explain the pattern of variation observed for *Ny. khinzir* at TM.

### *Nyanzachoerus khinzir* nov. sp. and the diversity of *Nyanzachoerus*

*Nyanzachoerus khinzir* presents a unique combination of features. For example, its third molars are as complex and proportionally as long as in *Ny. australis*, but they are as low-crowned as and closer in absolute length to those of *Ny. tulotos*. *Nyanzachoerus khinzir* is intermediate to these species for many other features, and does not encompass their combined ranges of variation. The species with the closest size and morphology to those of *Ny. khinzir* is *Ny. tulotos*.

Within the latter species, *Ny. khinzir* compares more closely with the late representatives from the Upper Nawata and the Adu-Asa. Similarities rely essentially on dental metrics with a moderate reduction of premolars in late *Ny. tulotos* compared to earlier representatives (see, notably, Fig. 7C). This is unsurprising, premolar reduction and third molar elongation being a rampant evolutionary trend in Tetraconodontinae as well as in Suinae [1], [2], [4]. In addition, Haile-Selassie ([10]: 345) suggested that some of the largest specimens of *Ny. tulotos* at Lothagam, from Upper Nawata and the Apak Member of the Nachukui Formation, could in fact belong to *Ny. australis*. Such restriction of the hypodigm of *Ny. tulotos* would be in favor of a greater distinction between this species and *Ny. khinzir*.

Within late specimens of *Ny. tulotos*, the most complete specimen, male cranium STD-VP-1/1 from Adu-Asa, differs in some noticeable aspects from the male cranium KNM-LT 316 from the Lower Nawata, possibly somewhat affected by postmortem distortion of both specimens. These differences bear essentially on dimorphic characters (notably expansion of zygomatic arches). They could be linked to the biological age and the sexual status of these specimens, KNM-LT 316 being an aged hypermale and STD-VP-1/1 a young adult. We observed such differences in extant species of suids, and they do not necessarily document any evolutionary trend or interspecific differentiation relevant to distinguish *Ny. khinzir* from ‘late’ *Ny. tulotos*.

The difference between *Ny. khinzir* on the one hand, and *Ny. australis* and *Ny. kanamensis* on the other hand, is notably expressed by the two latter species being larger, having third molars longer and much higher-crowned, generally lacking p1, and having proportionally smaller premolars. The difference between *Ny. australis* and *Ny. kanamensis* is much less conspicuous, and some authors propose alternately a placement of the material from Langebaanweg within *Ny. kanamensis* as a subspecies, *Ny. kanamensis australis* [1], [15], [38]. The Langebaanweg nyanzachoere has now been identified in deposits of Lothagam [17] and the Middle Awash research area [10] roughly contemporaneous to those of Langebaanweg.

Our observations confirm the initial diagnosis proposed by Cooke and Hendey [15], *Ny. australis* differing from *Ny. kanamensis* by: longer canine-second premolar diastemata; the consistent retention of P1; and overall larger tooth dimensions. In addition, *Ny. australis* has markedly more elongate p2 (Table S3). In the hypodigm of *Ny. kanamensis*, only specimens from the Ibole Member of the Wembere-Manonga Formation display similar dimensions [39]. When excluding these specimens, the difference is more pronounced, *Ny. kanamensis* displaying ranges of 7.0 mm–10.8 mm (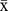 = 8.6 mm, N = 13) for p2 length and of 67.7–109.6 (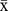 = 89.5, N = 13) for p2 width/length ratio (see Tables 4 and S3 for comparison). On the contrary, we did not observe marked differences of premolar size relative to molar size between *Ny. australis* and *Ny. kanamensis*.

The absence of a median conulid between the third pair of cuspids and the distoconid of m3 is viewed by Haile-Selassie [10] as a strong diagnostic feature of *Ny. australis*. First, in *Ny. kanamensis*, this conulid is absent not only from some Hadar specimens [10], but also from half a dozen Kanapoi specimens, including KNM-KP 239. Second, it is observed, sometimes unilaterally, in some specimens of *Ny. australis*: at Langebaanweg, on SAM-PQL 11889, SAM-PQL 14431, and SAM-PQL 20490; in the Kuseralee Member of the Sagantole Formation, on AMW-VP-1/21, KUS-VP-1/67, KUS-VP-1/102, as well as on AMW-VP-1/37, attributed to *Ny*. cf. *kanamensis* by Haile-Selassie [10] on the basis of the presence of the conulid. The occurrence of this conulid seems thus to display some degree of variation, being accounted as rare in *Ny. australis* whereas frequent in *Ny. kanamensis*.

Given these morphological differences, given that *Ny. australis* encompasses the entire distribution range of *Ny. kanamensis*, and given that it predates the first occurrence of *Ny. kanamensis* “sensu stricto” in the Middle Awash deposits ([10]: 358), we are in favor of the specific differentiation of *Ny. australis* and *Ny. kanamensis*. We further note that the material from the older deposits at Manonga Valley could possibly be better accommodated within *Ny. australis*, in good agreement with the relative large dimensions of the Manonga specimens as well as with the proposed temporal range of the Ibole Member [56].

The specimens found in south TM and attributed to *Ny*. cf. *australis* are homogeneously more advanced than *Ny. khinzir*, notably in their third molar complexity, hypsodonty, and premolar/molar ratios. Given the lack of more complete specimen, notably cranial, we viewed the attribution of this material to *Ny. australis* as likely but not certain, because it bears mostly on the development of the p2 evidenced only by alveoli or broken roots. Our recognition of this taxon in Chad is therefore provisional.

*Nyanzachoerus devauxi* is generally considered as the smallest species of the genus, with the simpler and shorter third molars (see, e.g. [10], [17], [31]). Unlike in the Oued el Hammam Valley where the holotype was found isolated [11], the presence of *Ny. devauxi* concurs with that of *Ny. syrticus* at Sahabi, and with that of *Ny. tulotos* at Lothagam and in the Adu-Asa Formation. At TM, no third molar combines the small size and the low CS observed in *Ny. devauxi*, and the overlap with *Ny. khinzir* is negligible. It is more complicated to discriminate with certainty large third molars of *Ny. devauxi* from small ones of *Ny. tulotos*, as they display quite similar morphological ranges (Fig. 6C, D). Similarly, premolars cannot be easily discriminated (Tables 3–6, Fig. 7). In contrast, the proportions of m1 relative to m3 and dental row relative lengths (Fig. 7B and Table 2) distinguish the species, so species determinations based only on isolated teeth may need further scrutiny. This is the case for the Adu-Asa Formation, where *Ny. devauxi* is represented by isolated unerupted molars and by a relatively small distal portion of a M3, ASK-VP-3/115, with a CS of 1.2 [10]. The distocone of this specimen has a height of 18.2 mm, i.e. much higher than other specimens of *Ny. devauxi* (Table 7), and could be well accommodated within *Ny. tulotos*. If the presence of *Ny. devauxi* in the Adu-Asa was to be revised on these grounds, the only well-dated occurrence of the species would be the Nawata Formation. To complicate the matter, it is to be noted that the holotype mandible was reported lost ([57]: 72) and recent efforts accomplished to find it did not succeed.

Although not documented by abundant material, *Ny. waylandi* is clearly distinct from *Ny. khinzir* in its flatter symphysis [10], [37], its absolutely smaller premolars (Tables 4–6), its lower premolars relatively narrower (Table S3), and its m3 short but on average relatively as narrow as in *Ny. australis* and *Ny. kanamensis* (Table 8). In the Kuseralee Member of the Sagantole Formation, *Ny. waylandi* co-occurs with *Ny. kuseralensis* Haile-Selassie, 2009 [10]. This species was principally distinguished from other species of *Nyanzachoerus* by its smaller size, its buccolingually compressed p3, its robust but shallow mandibular corpus, its higher premolar/molar ratio, its small m3 with two pairs of pillars, and its lack of p1 and p2 [10].

On the basis of our measurements, the holotype specimen of *Ny. kuseralensis*, KUS-VP-1/15, presents a small absolute size (Md5 = 123 mm, Table 2) not different from the range of *Ny. devauxi* and falling close to the same measurement in another specimen from the Kuseralee Member, AMW-VP-1/71 (Md5 = 139 mm, Table 2), attributed to *Ny. waylandi* by Haile-Selassie [10]. The p3 absolute dimensions and width/length ratio of KUS-VP-1/15 fall exactly within the ranges of *Ny. waylandi* (Tables 5 and S3). Corpus height at p2–p3 and corpus width across m1 are 49 mm and 39 mm respectively, not departing much from the values of AMW-VP-1/71 (ca. 52 mm and 36 mm respectively), both falling close to the lowest dimensions observed on specimens of Lower Nawata *Ny. tulotos* and Kanapoi *Ny. kanamensis*. We failed to find the premolar/molar ratio for KUS-VP-1/15 higher than those of all other species of *Nyanzachoerus*. On the contrary, its ratios tend to be similar to those of *Ny. kanamensis*, *Ny. australis*, and the high values observed for *Ny. khinzir* (see, e.g., Fig. 7C–D). The m3 of KUS-VP-1/15 present a talonid development also encountered in *Ny. khinzir* and correspond to a CS of 2.25 (Fig. 5M). Given the high degree of variation of the m3 talonid in extant *Potamochoerus* and in *Nyanzachoerus*, this morphology should not be considered incompatible with that observed in *Ny. waylandi* from the Kuseralee Member, with CS of 2.0 and 2.5 for AMW-VP-1/7 and AMW-VP-1/71, respectively.

We failed to find any significant departure in measurements and dental ratios of the specimens attributed to *Ny. waylandi* and *Ny. kuseralensis* in the Kuseralee Member (see, e.g., Fig. 7B). A single feature seemed to differ radically between AMW-VP-1/71 and KUS-VP-1/15: the complete lack of p2 in the latter. In the extant sample of *Potamochoerus* we observed, the presence of p2 is variable: out of 48 specimens of *P. porcus*, it was absent bilaterally in one case, and twice unilaterally; out of 76 specimens of *P. larvatus*, it was absent bilaterally in no less than 12 specimens, and unilaterally in seven. Similarly, in *Ny. khinzir*, we found no p2 alveoli in two specimens, including above discussed TM 170-01-019. Given the variations observed in extant species, it seems more cautious to not consider the presence/absence of the reduced mesial premolars of *Nyanzachoerus* as an absolutely diagnostic feature, other than referring to frequency differences based on relatively large samples. We therefore failed to distinguish KUS-VP-1/15 from *Ny. waylandi*, and therefore consider *Ny. kuseralensis* as a junior synonym of the latter.

Finally, *Ny. khinzir* markedly differs from *Ny. jaegeri*, which is larger and has significantly higher-crowned, longer and more complex third molars, proportionally smaller premolars, and a more sexually dimorphic craniomandibular morphology reminiscent of more derived African tetraconodonts (see notably [4], [8], [14], [17]). The generic attribution of *Ny. jaegeri* is currently debated, with a proposal to attribute this species to *Notochoerus* ([34]; but see [58]).

### Paleoecology of *Nyanzachoerus khinzir* nov. sp

**Figure 8:**
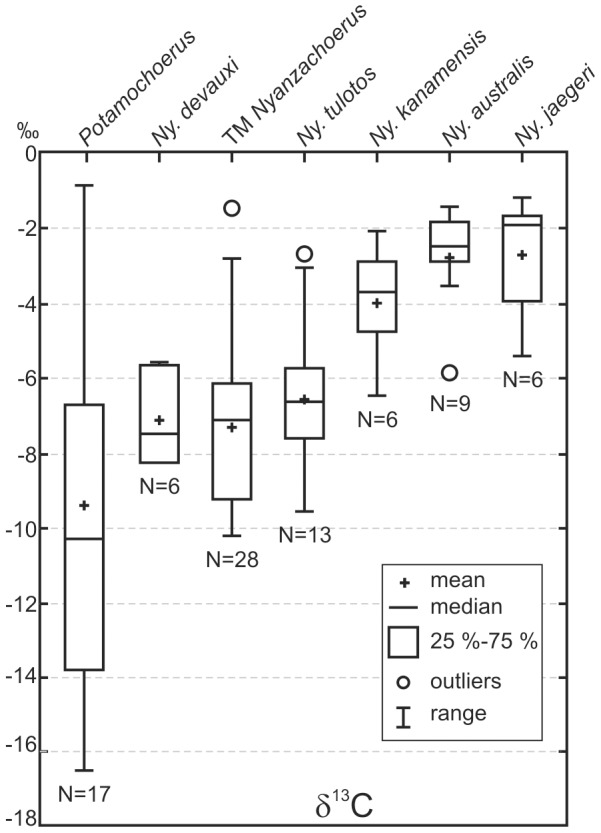
δ^13^C values (in ‰) for *Nyanzachoerus* from TM and from the Turkana Basin. TM data are from TM 266 and TM 267. Extant comparative data (*Potamochoerus*) are from [51]. Data for eastern African *Nyanzachoerus* are from [51], [59], [60].

*Nyanzachoerus khinzir* is morphologically close to *Ny. tulotos* of eastern Africa [10], [14], [17], [42]. Their dental morphological proximity implies that these species may have been ecologically similar. Carbon stable isotope results from TM 266-TM 267 support this view, indicating a diversified diet including a mixture of C_3_ and C_4_ plants (Fig. 8, Table S6) as for *Ny. tulotos* from the Turkana Basin [51], [59], [60]. Although TM *Nyanzachoerus* displays an average diet slightly more C_3_-enriched than *Ny. devauxi* and *Ny. tulotos* and much more C_3_-enriched than other specimens of *Nyanzachoerus* from the Turkana Basin, some TM individuals had a diet quite rich in C_4_ plants (being most generally grasses). It can be noted that no sample of *Nyanzachoerus* displays a range of variation similar to what is observed for extant *Potamochoerus* (Fig. 8). This could be interpreted as an indication of more constant food preferences than in *Potamochoerus*, but it may also reflect a poor representation of populations that lived in closed habitats in late Miocene–early Pliocene sites.

TM *Nyanzachoerus* has low values of δ^18^O within the TM fauna: between −9.0 ‰ and 0.5 ‰, with a mean value of −3.7 ‰ for 27 specimens (Table S6). They match only those of local hippopotamids and anthracotheriids [21]. The average values are much lower than those obtained for *Nyanzachoerus* from the Turkana Basin, ranging from −4.9 ‰ to 1.4 ‰ with a mean of −1.3 ‰ for 24 specimens (data compiled from [51], [59]). This possibly indicates that *Nyanzachoerus* was particularly dependent on wet habitats at TM 266-TM 267.

Therefore, the occurrence of *Nyanzachoerus* at TM differed from that at Lothagam in diversity (only one species at TM, two to three in the Nawata Formation [17]) as well as in stable isotopic data. This may be linked to different environmental settings: the Lothagam ecosystem was identified as a dwarf shrubland [59] whereas that of TM was previously described as analogous to the Okavango Delta in central Kalahari, Botswana [61].

### Paleobiogeographical implications

**Figure 9:**
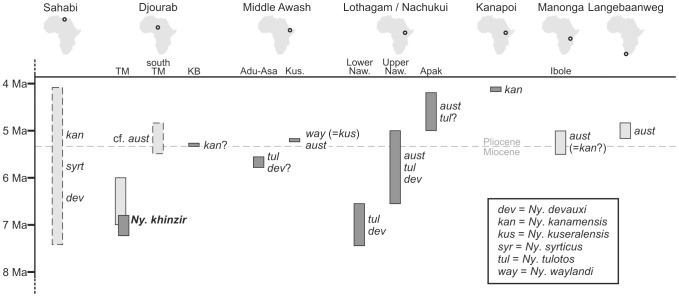
Geographical and temporal distribution of the main sites and species of *Nyanzachoerus* compared with *Ny. khinzir* nov. sp. Abbreviated sites are: TM, Toros-Ménalla; south TM, south Toros-Ménalla; KB, Kossom Bougoudi; Kus., Kuseralee Member of the Sagantole Formation; Lower Naw., Lower Member of the Nawata Formation; Upper Naw., Upper Member of the Nawata Formation; Ibole, Ibole Member of the Wembere-Manonga Formation. Vertical bars represent site temporal range: light tone, biochronologically dated ranges; dark tone, geochronologically dated ranges; discontinuous outline, uncertain ranges. Biochronological range for TM is from [19], geochronological ranges for TM and Kossom Bougoudi are from [29].

Previous interpretations of the suid material from Sahabi and TM [19], [31] suggested a wide distribution of the late Miocene *Ny. syrticus* encompassing northern, central, and eastern Africa. Our findings suggest a more fragmented geographical distribution for species of *Nyanzachoerus* during this time interval: *Nyanzachoerus khinzir* is so far known only from the TM localities in central Africa; *Ny. tulotos* is so far known only from eastern Africa (Fig. 9).

The ‘anthracotheriid unit’ further differs from other localities of roughly this antiquity in lacking the small *Ny. devauxi*, found in northern Africa [11], [31], [33] and eastern Africa [10], [17]. Other TM taxa previously indicated a faunal provincialism of the Lake Chad Basin with regard to eastern Africa (e.g., anthracotheriids, hippopotamids, bovids, birds, carnivores, fishes, see [20], [23], [24], [62]–[64] respectively), as well as stronger affinities with the Sirte Basin in Libya, and beyond with Eurasia. The specific distribution of *Nyanzachoerus* is therefore congruent with a biogeographical distinction between central Africa and eastern Africa. The biogeographical affinities of the late Miocene *Nyanzachoerus* from northern Africa are more uncertain because of limited samples, fragmentary preservation, and stratigraphic uncertainties.

### Biochronological implications

Together with other taxa, the tetraconodont material of TM was initially used to correlate the ‘anthracotheriid unit’ to the Lower Member of the Nawata Formation at Lothagam [19]. This was done on a limited sample confined to locality TM 266 and since then has been considerably augmented for a variety of mammalian taxa. The present work led us to recognize that this suid assemblage is instead attributable to a new species not yet found in other African sites, and therefore not supportive of previous correlation. Currently, it is therefore not possible to propose a biochronological correlation of TM on the basis of this suid material. The previous biochronological correlation proposed by Vignaud et al. ([19]: 154) between the ‘anthracotheriid unit’ and the Lower Nawata deposits remains supported by other faunal elements (prominently the proboscideans), the age of TM being based on this correlation and on the radiochronological dates proposed by Lebatard et al. [29], [30]. The temporal placements of *Nyanzachoerus* from TM and other important Mio-Pliocene sites are summarized in Figure 9.

Within the ‘anthracotheriid unit’, locality TM 9 is somewhat different from other localities bearing *Ny. khinzir* because of specimen TM 9-01-019. This specimen could come from an unusual individual, which presence is not unexpected in such a large sample. Alternately, it could be indicative of an environmental and/or chronological difference between TM 9 and other localities. TM 9 is further particular because it is the richest TM locality with so few *Libycosaurus* (less than 1% of ca. 800 collected specimens). A chronological lag between TM 9 and other TM localities would probably involve limited time, because TM 9 otherwise differs clearly from Chadian fossiliferous areas with later deposits including more advanced tetraconodonts and different suid associations, namely Kossom Bougoudi in the upper-most Miocene [65], [66] and Kollé in the lower Pliocene [67].

A subset of TM localities (south TM) bears a different, more advanced tetraconodont, provisionally recognized as *Ny*. cf. *australis*. According to the First Appearance Datum proposed for *Ny. australis* by Haile-Selassie [10] using the record of the Middle Awash research area in Ethiopia, this could indicate that these Chadian sediments were deposited after 5.5 Ma. South TM is distinct from the rest of TM localities in bearing the camelid *Paracamelus gigas*, whereas this taxon is present at Kossom Bougoudi [68] where it constitutes the oldest known occurrence of a camelid in Africa, dated to the Mio-Pliocene transition [29], [65]. It also lacks any remains of *Libycosaurus*. Further work should be necessary to determine if south TM could be coeval with Kossom Bougoudi (Fig. 9), notably a detailed comparison between *Ny*. cf. *australis* and the tetraconodont material of Kossom Bougoudi, initially attributed to *Ny. kanamensis* [65].

## Supporting Information

Figure S1 **Dental nomenclature after** [47] **.** A, tooth orientation: the top arrow indicates the mesio-lingual direction; B, structures identifiable on cingula/-ids; C, sketch of a right upper molar in occlusal view; D, Sketch of a right lower molar in occlusal view; E, sketch of a right upper premolar in occlusal view; F, Sketch of a right lower premolar in occlusal view. Abbreviations: Para., paracone; Proto., protocone; Meta., metacone; Metaul., metaconule; Metad., metaconid; Protod., protoconid; Entod., entoconid; Hypod., hypoconid; Hypoulid., hypoconulid; -c., -crista; -cul., -cristule; -cid., -cristid; -culid., -cristulid.
(TIF)

Figure S2 **Complexity scoring of third molar variations in Nyanzachoerus.** Variation ranges of complexity scores (CS) for: A, talons of upper third molars and B, talonids of lower third molars, mapped from various specimens of Nyanzachoerus. Grey cusps/-ids are not considered in the definition of the CS.
(TIF)

Table S1 Additional cranial measurements (min.-max. in mm; mean; N) in Nyanzachoerus. Abbreviations: LN, Lower Nawata; AA, Adu-Asa; LW, Langebaanweg; *, male; Cr6, length between rostral extremity of premaxillae and staphylion; Cr7, length between staphylion and basion; Cr8, length between rostral and nuchal extremities of premaxillae; Cr9, premaxilla width at I3 level; Cr10, width between medial edges of canine alveoli; Cr11, width between P2 mesial extremities; Cr12, width between M3 distal extremities; Cr13, maximal enlargement of nasals on dorsal side, above premolar rows; Cr14, height between dorsal edge of orbit and M3 distal extremity.
(PDF)

Table S2 Additional mandibular measurements (min.-max. in mm; mean; N) in Nyanzachoerus. Abbreviations: LN, Lower Nawata; UN, Upper Nawata; AA, Adu-Asa; LW, Langebaanweg; *, male; a, includes other specimens from Sahabi described as Ny. cf. syrticus by Cooke [31] in addition to the holotype; Md6, length between i1-i1 diastema and nuchal extremity of condyle; Md7, length between i1-i1 diastema and nuchal extremity of symphysis; Md8, width between distal i3; Md9, width between mesial p2; Md10, width between lingual p4; Md11, c-p2 diastema; Md12, c-p3 diastema.
(PDF)

Table S3 Premolar width/length ratios (min.-max., mean; N) in Nyanzachoerus. Abbreviations: LN, Lower Nawata; UN, Upper Nawata; AA, Adu-Asa; LW, Langebaanweg; K, Kanapoi; M, Manonga; a, includes other specimens from Sahabi described as cf. Ny. syrticus by Cooke [31] in addition to holotype; difference with sample of Ny. khinzir (t-test): °, non-significant; *, p<0.05; **, p<0.01; ***: p<0.001. For all premolars, ratio is 100*w/L.
(PDF)

Table S4 Additional molar measurements (min.-max. in mm; mean; N) in Nyanzachoerus. Abbreviations: LN, Lower Nawata; UN, Upper Nawata; AA, Adu-Asa; LW, Langebaanweg; K, Kanapoi; L, mesiodistal length at cervix; w, labiolingual width of mesial lobe of crown; difference with sample of Ny. khinzir (t-test): °, non-significant; *, p<0.05; **, p<0.01; ***: p<0.001.
(PDF)

Table S5 Additional third molar measurements (min.-max. in mm; mean; N) in Nyanzachoerus. Abbreviations: LN, Lower Nawata; UN, Upper Nawata; AA, Adu-Asa; LW, Langebaanweg; h1, maximal height of the first pair of cusps/-ids; h2, maximal height of the second pair of cusps/-ids; H1, hypsodonty index (100*(h1/w)) of the first pair of cusps/-ids; H2, hypsodonty index (100*(h2/w)) of the second pair of cusps/-ids; H-tal, hypsodonty index (100*(h-tal/w)) of the talon/-id.
(PDF)

Table S6 Stable isotope data (δ13C and δ18O in ‰) obtained from carbonates of enamel apatite of Nyanzachoerus at TM 266 and TM 267. Abbreviations: *, specimens attributed to Ny. khinzir nov. sp. (other specimens are attributed to Nyanzachoerus sp.).
(PDF)

Table S7 Craniodental measurements (in mm) and complexity scores of TM Nyanzachoerus. Note: measurements obtained with a digital caliper with a maximum deviation of 0.02 mm, rounded to nearest 0.1 mm.
(XLS)

Text S1 **Specimens of Nyanzachoerus from Toros-Ménalla, Chad.**
(PDF)

Text S2 **Summary of the dental nomenclature.**
(PDF)

Text S3 **Definition of complexity scores (CS).**
(PDF)
