# Supplementary figures and images for: A New Species of Nyanzachoerus (Cetartiodactyla: Suidae) from the Late Miocene Toros-Ménalla, Chad, Central Africa
Source: PLoS One. 2014 Aug 27;9(8):e103221. doi: 10.1371/journal.pone.0103221 (PMC4146473; doi:10.1371/journal.pone.0103221)

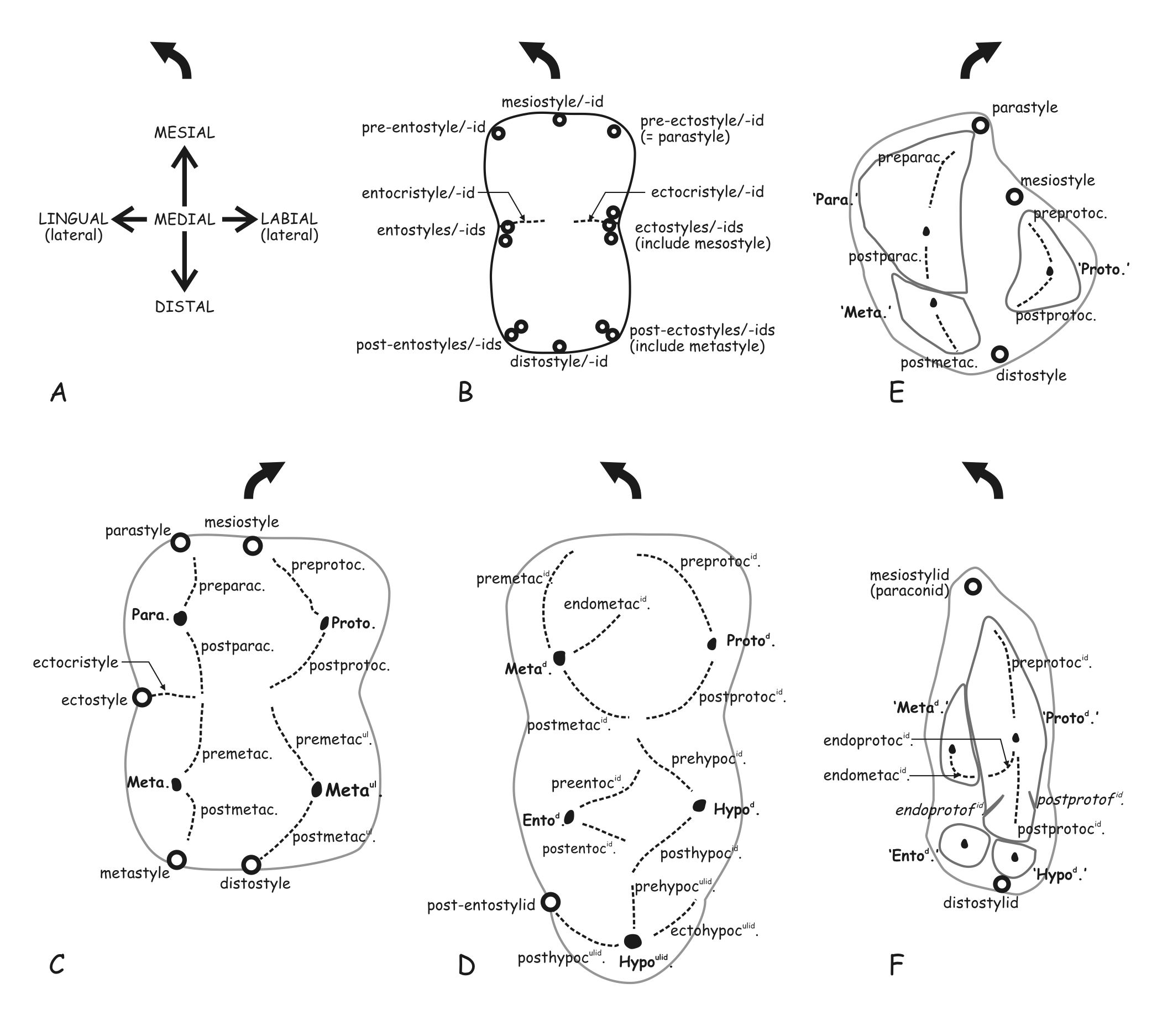

Supplement: Figure S1 — Dental nomenclature after [47] . A, tooth orientation: the top arrow indicates the mesio-lingual direction; B, structures identifiable on cingula/-ids; C, sketch of a right upper molar in occlusal view; D, Sketch of a right lower molar in occlusal view; E, sketch of a right upper premolar in occlusal view; F, Sketch of a right lower premolar in occlusal view. Abbreviations: Para., paracone; Proto., protocone; Meta., metacone; Metaul., metaconule; Metad., metaconid; Protod., protoconid; Entod., entoconid; Hypod., hypoconid; Hypoulid., hypoconulid; -c., -crista; -cul., -cristule; -cid., -cristid; -culid., -cristulid. (TIF) [file pone.0103221.s001.tif]

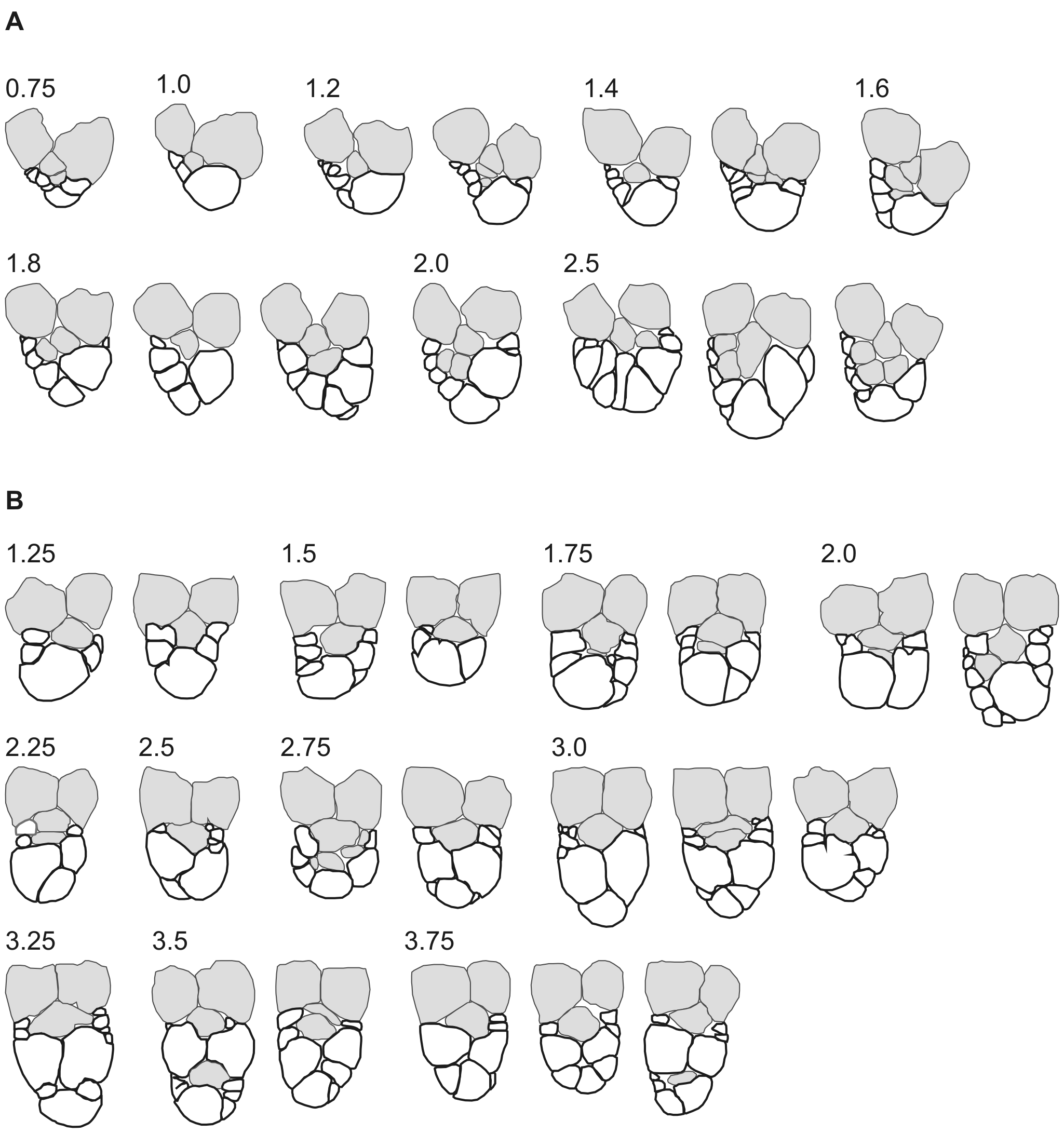

Supplement: Figure S2 — Complexity scoring of third molar variations in Nyanzachoerus. Variation ranges of complexity scores (CS) for: A, talons of upper third molars and B, talonids of lower third molars, mapped from various specimens of Nyanzachoerus. Grey cusps/-ids are not considered in the definition of the CS. (TIF) [file pone.0103221.s002.tif]
