# Supplementary material for: A New Species of Nyanzachoerus (Cetartiodactyla: Suidae) from the Late Miocene Toros-Ménalla, Chad, Central Africa
Source: PLoS One. 2014 Aug 27;9(8):e103221. doi: 10.1371/journal.pone.0103221 (PMC4146473; doi:10.1371/journal.pone.0103221)
Supplement: Table S1 — Additional cranial measurements (min.-max. in mm; mean; N) in Nyanzachoerus. Abbreviations: LN, Lower Nawata; AA, Adu-Asa; LW, Langebaanweg; *, male; Cr6, length between rostral extremity of premaxillae and staphylion; Cr7, length between staphylion and basion; Cr8, length between rostral and nuchal extremities of premaxillae; Cr9, premaxilla width at I3 level; Cr10, width between medial edges of canine alveoli; Cr11, width between P2 mesial extremities; Cr12, width between M3 distal extremities; Cr13, maximal enlargement of nasals on dorsal side, above premolar rows; Cr14, height between dorsal edge of orbit and M3 distal extremity. (PDF) [file pone.0103221.s003.pdf]

Table S1. Additional cranial measurements (min.-max. in mm; mean; N) in *Nyanzachoerus*.

Taxa		Cr6	Cr7	Cr8	Cr9	Cr10	Cr11	Cr12	Cr13	Cr14	100*Cr2 / Cr5	100*Cr6 / Cr5
TM: *Ny. khinzir*	all	305	97-107; 102.0; 3	79-88; 83.0; 3	64-72; 68.3; 4	53-77; 63.3; 4	51-61; 55.6; 9	47-65; 56.1; 15	73-80; 75.3; 3	137-157; 148.6; 5	23.5-35.1; 28.0; 12	34.3-37.9; 36.1; 2
	males		97		72	67-77; 72.0; 2	51-61; 57.0; 4	60	73-80; 75.3; 3	154-155; 154.5; 2	27.7-30.5; 29.1; 2	37.9
	females	305	102	88	72	53	56	59		140	24.6	
Ny. tulotos	all	268-348; 311.3; 3	90-108; 101.7; 3	65-96; 82.7; 3	61-80; 71.0; 3	48-59; 55.0; 3	45-52; 49.3; 3	47-63; 56.3; 3	67-67; 67.0; 2	145-151; 148.0; 2	21.2-24.4; 22.9; 3	29.2-32.8; 30.6; 3
	LN	268-318; 293.0; 2	90-108; 99.0; 2	65-87; 76.0; 2	61-72; 66.5; 2	48-58; 53.0; 2	45-51; 48.0; 2	47-63; 55.0; 2	67-67; 67.0; 2	145-145; 145.0; 1	21.2-24.4; 22.8; 3	29.2-32.8; 31.0; 2
	AA	348*	107*	96*	80*	59*	52*	59*		151*	23.2	29.7
Ny. australis	all	343-367; 355.0; 2	115	80-102; 89.3; 4	90-90; 90.0; 2	93-93; 93.0; 2	55-67; 61.2; 3	61-76; 68.1; 3			26.5-29.3; 28.1; 3	
	LW	343-367; 355.0; 2	115	80-102; 89.3; 4	90-90; 90.0; 2	93-93; 93.0; 2	62-67; 64.5; 2	67-76; 71.5; 2			26.5-29.3; 27.2; 2	
	AA						55	61			28.3	
Ny. kanamensis (Kanapoi)		284-354; 317.3; 3	126	70-101; 88.3; 4	74-86; 80.3; 4	58-74; 66.3; 3	47-82; 64.8; 4	54-59; 56.3; 3	69-74; 71.5; 2	106	28.5-29.9; 29.2; 2	
Ny. devauxi		265		67	60	45	46	48-50; 49.0; 2			24.8-27.3; 26.0; 2	

Abbreviations: LN, Lower Nawata; AA, Adu-Asa; LW, Langebaanweg; *, male; Cr6, length between rostral extremity of premaxillae and staphylion; Cr7, length between staphylion and basion;Cr8, length between rostral and nuchal extremities of premaxillae; Cr9, premaxilla width at I3 level;Cr10, width between medial edges of canine alveoli; Cr11, width between P2 mesial extremities; Cr12, width between M3 distal extremities; Cr13, maximal enlargement of nasals on dorsal side, above premolar rows; Cr14, height between dorsal edge of orbit and M3 distal extremity.
