# Supplementary material for: A New Species of Nyanzachoerus (Cetartiodactyla: Suidae) from the Late Miocene Toros-Ménalla, Chad, Central Africa
Source: PLoS One. 2014 Aug 27;9(8):e103221. doi: 10.1371/journal.pone.0103221 (PMC4146473; doi:10.1371/journal.pone.0103221)
Supplement: Table S2 — Additional mandibular measurements (min.-max. in mm; mean; N) in Nyanzachoerus. Abbreviations: LN, Lower Nawata; UN, Upper Nawata; AA, Adu-Asa; LW, Langebaanweg; *, male; a, includes other specimens from Sahabi described as Ny. cf. syrticus by Cooke [31] in addition to the holotype; Md6, length between i1-i1 diastema and nuchal extremity of condyle; Md7, length between i1-i1 diastema and nuchal extremity of symphysis; Md8, width between distal i3; Md9, width between mesial p2; Md10, width between lingual p4; Md11, c-p2 diastema; Md12, c-p3 diastema. (PDF) [file pone.0103221.s004.pdf]

Table S2. Additional mandibular measurements (min.-max. in mm; mean; N) in *Nyanzachoerus*.

Taxa		Md6	Md7	Md8	Md9	Md10	Md11	Md12	100*Md3 / Md5
TM: *Ny. khinzir*	all	394	105-154; 123.8; 12	52-74; 62.4; 11	39-60; 48.9; 8	36-46; 41.7; 6	44-62; 51.0; 15	56-75; 64.4; 15	29.7-36.4; 33.3; 27
	males		123-154; 135.5; 4	55-74; 66.8; 5	39-60; 48.7; 3	38-43; 40.5; 2	47-62; 55.1; 4	67-75; 70.6; 4	33.0-36.1; 34.8; 4
	females	394	105-118; 113.3; 3	52-60; 54.7; 3	46-50; 47.7; 3	36-46; 41.3; 3	44-54; 48.9; 6	56-70; 62.3; 6	32.1-36.4; 33.2; 6
TM: *Ny.* cf. *australis*							48	63	31.7-36.2; 33.5; 4
Ny. tulotos	all		93-144; 118.5; 2	44-61; 54.3; 3	42-46; 43.5; 4	30-38; 34; 2	27-52; 40.8; 9	38-67; 54.5; 9	35.1-38.8; 37.1; 8
	LN		93	44-58; 51.0; 2	42-46; 44.0; 3	30-38; 34.0; 2	27-46; 39.4; 8	38-61; 53.1; 8	35.5-38.8; 37.4; 7
	UN		144	61	42		52	67	35.1
Ny. syrticus			141*	74*	68*	48*	55*	70*	34.9*
Nyanzachoerus sp. from Sahabi						36			
Ny. australis	all	414	132	72-78; 75.0; 2	60-64; 62.4; 4	49-63; 53.8; 3	53-66; 61.1; 4	68-79; 74.5; 4	28.4-33.6; 31.2; 10
	LW	414	132	72-78; 75.0; 2	61	49	60-66; 63.0; 2	74-79; 76.5; 2	28.4-31.7; 30.2; 4
	AA				60-64; 62.9; 3	50-63; 56.3; 2	53-66; 59.2; 2	68-77; 72.5; 2	30.2-33.6; 31.8; 6
Ny. kanamensis (Kanapoi)		399	117-148; 135.0; 3	54-65; 59.4; 5	58-79; 66.3; 4	44-54; 50.3; 3	46-53; 50.0; 4	56-67; 59.8; 4	31.3-35.0; 33.7; 7
Ny. devauxi			110						34.1-35.3; 34.7; 2
Ny. waylandi							46		34.5
Ny. kuseralensis			94	68		41		57	32.4

Abbreviations: LN, Lower Nawata; UN, Upper Nawata; AA, Adu-Asa; LW, Langebaanweg; *, male; Md6, length between i1-i1 diastema and nuchal extremity of condyle; Md7, length between i1-i1 diastema and nuchal extremity of symphysis; Md8, width between distal i3; Md9, width between mesial p2; Md10, width between lingual p4; Md11, c-p2 diastema; Md12, c-p3 diastema.
