# Supplementary material for: A New Species of Nyanzachoerus (Cetartiodactyla: Suidae) from the Late Miocene Toros-Ménalla, Chad, Central Africa
Source: PLoS One. 2014 Aug 27;9(8):e103221. doi: 10.1371/journal.pone.0103221 (PMC4146473; doi:10.1371/journal.pone.0103221)
Supplement: Table S3 — Premolar width/length ratios (min.-max., mean; N) in Nyanzachoerus. Abbreviations: LN, Lower Nawata; UN, Upper Nawata; AA, Adu-Asa; LW, Langebaanweg; K, Kanapoi; M, Manonga; a, includes other specimens from Sahabi described as cf. Ny. syrticus by Cooke [31] in addition to holotype; difference with sample of Ny. khinzir (t-test): °, non-significant; *, p<0.05; **, p<0.01; ***: p<0.001. For all premolars, ratio is 100*w/L. (PDF) [file pone.0103221.s005.pdf]

Table S3. Premolar width/length ratios (min.-max., mean; N) in *Nyanzachoerus*.

Taxa		P1	P2	P3	P4	p1	p2	p3	p4
TM: *Ny. khinzir*		57.6-63.6; 60.6; 2	54.3-71.3; 59.8; 6	76.3-102.2; 86.6; 32	106.6-138.5; 124.1; 38	58.3	46.3-81.5; 62.5; 11	67.5-90.6; 79.9; 35	88.5-109; 95.8; 46
TM: *Ny.* cf. *khinzir*			64.8		127.5				
TM: *Ny.* cf. *australis*				81.6	129.3			78.6-80.3; 79.5; 2	93.3-99.1; 96.2; 2
Ny. tulotos	all	54.0-54.2; 54.1; 2	56.9-65.0; 61.2; 4	75.3-107; 94.1; 12	107.1-140.1; 125.3; 22°	48.6-60.8; 53.3; 3	44.7-63.8; 55.5; 9	73.0-94.8; 84.4; 21**	83.3-111.4; 93.2; 28°
	LN	54.2	64.7-65; 64.9; 2	93.2-105.9; 97.6; 5	117.3-140.1; 128.9; 10	48.6-50.4; 49.5; 2	48.2-63.8; 57.1; 7	79.6-94.8; 86.8; 10	84.4-110.4; 94.0; 17
	UN		58.0	93.1	115.7-135.5; 126; 3	60.8	44.7-54.5; 49.6; 2	73-94.2; 85; 7	111.4
	AA	54.0	56.9	75.3-94.2; 85.8; 4	121.1-124.1; 122.8; 5			80.2	83.3-94.5; 89.2; 6
Ny. syrticus						58.1	64.3	84.3	88.2
Nyanzachoerus sp. from Sahabi									104.8
Ny. australis	all	55.8-72.2; 64.0; 4	50.0-70.8; 60.5; 10	78.5-103.5; 89.7; 26°	110.3-136.8; 122.2; 26°		56.6-75; 61.8; 15	64.1-88.6; 77.9; 29°	82.2-106.9; 93.8; 34°
	LW	55.8-72.2; 64.0; 4	50.0-70.8; 60.4; 8	78.5-103.5; 90.4; 15	110.4-136.8; 121.7; 14		57.2-75; 63.4; 9	73.6-88.6; 81.7; 15	85.1-106.9; 95.6; 18
	AA		54.8-67.0; 60.9; 2	80.4-100.0; 87.5; 10	110.3-133.0; 121.2; 10		56.6-64.0; 59.3; 6	68.6-88.1; 74.4; 12	82.2-100.0; 91.7; 15
Ny. kanamensis	all		55.8-93.5; 69.0; 8	76.5-111.6; 94.5; 20***	113.2-147.1; 124.9; 19		53.5-109.6; 85.1; 15	51.7-94.6; 80.7; 32°	68.4-102.3; 90.9; 41**
	K		55.8-70.2; 62.6; 3	76.5-105.9; 88.6; 5	116.9-130.2; 120.6; 5		67.7-107.1; 81.2; 5	71.0-92.9; 79.5; 10	77.3-99.6; 91.4; 12
	M		64.0-69.1; 66.5; 2	87.3-104.2; 98.3; 6	118.5-128.9; 123.9; 4		53.5-59.3; 56.4; 2	94.4	86.7-98.1; 92.4; 2
Ny. devauxi		46.1-55.8; 50.9; 2	47.5-62; 54.8; 2	78.6-88.7; 82.1; 4	115.9-128.9; 123.7; 3	49.5	43.7-60; 51.8; 2	67.5-89.1; 77.7; 5	81.0-98.0; 91.5; 8
Ny. waylandi				83.2	115.5-125.6; 120.6; 2		47.3	57.1-71.0; 64.1; 2	66.7-79.7; 75.2; 3
Ny. kuseralensis								68.8	75.9

Abbreviations: LN, Lower Nawata; UN, Upper Nawata; AA, Adu-Asa; LW, Langebaanweg; K, Kanapoi; M, Manonga; difference with sample of *Ny. khinzir* (t-test): °, non-significant; *, $p<0.05$; **, $p<0.01$; ***: $p<0.001$. For all premolars, ratio is 100*w/L.
