# Supplementary material for: A New Species of Nyanzachoerus (Cetartiodactyla: Suidae) from the Late Miocene Toros-Ménalla, Chad, Central Africa
Source: PLoS One. 2014 Aug 27;9(8):e103221. doi: 10.1371/journal.pone.0103221 (PMC4146473; doi:10.1371/journal.pone.0103221)
Supplement: Table S4 — Additional molar measurements (min.-max. in mm; mean; N) in Nyanzachoerus. Abbreviations: LN, Lower Nawata; UN, Upper Nawata; AA, Adu-Asa; LW, Langebaanweg; K, Kanapoi; L, mesiodistal length at cervix; w, labiolingual width of mesial lobe of crown; difference with sample of Ny. khinzir (t-test): °, non-significant; *, p<0.05; **, p<0.01; ***: p<0.001. (PDF) [file pone.0103221.s006.pdf]

Table S4. Additional molar measurements (min.-max. in mm; mean; N) in *Nyanzachoerus*.

Taxa		L M1	w M1	L M2	w M2	L m1	w m1	L m2	w m2
TM: *Ny. khinzir*		16.8-21.5; 19.2; 35	17.8-23.3; 20.1; 28	22.9-29.0; 26.4; 40	21.6-28.6; 25.9; 36	16.5-22.4; 19.5; 54	13.3-20.2; 16.0; 37	22.7-32.1; 26.4; 72	18.6-25.0; 21.4; 56
TM: *Ny.* cf. *khinzir*		19.3	21.8	30.1	28.2				
TM: *Ny.* cf. *australis*		18.6-20.7; 19.7; 2	20.7	26.4	29.4	20.1-21.4; 20.8; 2	16.0	27.9-29.9; 28.6; 3	22.7
Ny. tulotos	all	17.2-25.1; 20.8; 12	18.9-23.1; 20.6; 9	22.4-30.0; 26.2; 20°	21.6-29.6; 26.2; 19	18.0-23.4; 19.5; 15	15.6-17.4; 16.5; 14	23.3-28.9; 25.5; 22^{*}	18.9-25.0; 21.6; 22°
	LN	18.2-25.0; 20.6; 6	18.9-21.4; 20.6; 5	24.0-28.0; 25.8; 12	21.6-28.0; 25.8; 12	18.1-20.6; 19.4; 9	15.6-17.2; 16.5; 8	23.3-28.3; 25.4; 15	19.7-24.1; 21.6; 14
	UN	17.2-25.1; 21.6; 4	19.2-23.1; 20.6; 3	24.9-30; 27.9; 3	24.0-29.6; 27.3; 3	18.0-23.4; 20.4; 3	16.2-17.4; 16.7; 3	23.4-28.9; 25.7; 4	19.2-25.0; 22.1; 4
	AA	20.1-20.2; 20.2; 2	20.8	26.5-27.4; 27.1; 4	26.1-27.4; 26.7; 3	18.9	15.7	25.3-27.0; 26.2; 2	18.9-21.9; 20.4; 2
Ny. syrticus						21.6	19.2	26.9	
Ny. australis	all	18.4-26.0; 21.5; 17	18.4-24.3; 21.3; 15	24.1-39.9; 30.7; 20^{***}	25.5-34; 29.2; 17	17.6-26.2; 20.8; 25^{***}	14.6-24.6; 17.3; 19	27.4-35.6; 30.5; 24^{***}	19.1-26.6; 22.9; 22^{***}
	LW	19.4-26.0; 22.3; 13	19.2-24.3; 22.0; 11	27.5-34.4; 31.1; 13	26.5-34.0; 30.0; 12	17.6-26.2; 21.5; 15	15.5-24.6; 17.8; 13	29.3-35.6; 31.5; 12	22.2-26.6; 24; 11
	AA	18.4-20.4; 19.2; 4	18.4-21.1; 19.4; 4	24.1-30.5; 27.2; 5	25.5-27.1; 26.1; 3	18.3-21.8; 19.7; 10	14.6-18.8; 16.2; 6	27.4-31.5; 28.9; 9	19.1-24.6; 21.7; 11
Ny. kanamensis	all	15.1-23.0; 19.2; 15	16.4-22.3; 18.9; 12	23.2-37.6; 29.5; 20^{***}	23.7-33.6; 26.9; 19	16.4-30.5; 19.9; 21°	12.0-20.0; 14.7; 20^{***}	22.6-31.9; 26.7; 36°	15.2-25.8; 20.8; 34°
	K	16.9-23.0; 20.2; 4	17.8-19.2; 18.5; 2	23.2-31.8; 28.0; 6	23.7-28.4; 25.1; 6	17.8-21.5; 20.0; 6	13.2-15.3; 14.2; 6	23.3-31.9; 26.8; 15	15.2-25.8; 21.1; 14
Ny. devauxi		17.2-21.9; 19.6; 2	17.6-18.7; 18.2; 2	23.0-25.0; 24.2; 5	23.0-24.8; 23.6; 5	16.3-20.1; 18.2; 8	13.3-18.0; 15.4; 8	19.6-26.5; 23.3; 11	16.8-20.6; 18.4; 11
Ny. waylandi				25.1-28.7; 26.9; 2	16.0	16.0-21.0; 19.1; 3	12.3-14.4; 13.6; 3	24.0-30.2; 27.4; 3	18.7-20.0; 19.2; 3
Ny. kuseralensis				22.7	22.6	16.5	13.8	23.7	

Abbreviations: LN, Lower Nawata; UN, Upper Nawata; AA, Adu-Asa; LW, Langebaanweg; K, Kanapoi; L, mesiodistal length at cervix; w, labiolingual width of mesial lobe of crown; difference with sample of *Ny. khinzir* (t-test): °, non-significant; *, $p<0.05$; **, $p<0.01$; ***: $p<0.001$.
