# Supplementary material for: A New Species of Nyanzachoerus (Cetartiodactyla: Suidae) from the Late Miocene Toros-Ménalla, Chad, Central Africa
Source: PLoS One. 2014 Aug 27;9(8):e103221. doi: 10.1371/journal.pone.0103221 (PMC4146473; doi:10.1371/journal.pone.0103221)
Supplement: Table S5 — Additional third molar measurements (min.-max. in mm; mean; N) in Nyanzachoerus. Abbreviations: LN, Lower Nawata; UN, Upper Nawata; AA, Adu-Asa; LW, Langebaanweg; h1, maximal height of the first pair of cusps/-ids; h2, maximal height of the second pair of cusps/-ids; H1, hypsodonty index (100*(h1/w)) of the first pair of cusps/-ids; H2, hypsodonty index (100*(h2/w)) of the second pair of cusps/-ids; H-tal, hypsodonty index (100*(h-tal/w)) of the talon/-id. (PDF) [file pone.0103221.s007.pdf]

Table S5. Additional third molar measurements (min.-max. in mm; mean; N) in *Nyanzachoerus*.

Taxa		h1 M3	h2 M3	H1 M3	H2 M3	H-tal M3	h1 m3	h2 m3	H1 m3	H2 m3	H-tal m3
TM: *Ny*. *khinzir*		19.3-21.2; 20.3; 2	17.2-18.6; 17.8; 4	63.5-69.5; 66.5; 2	56.9-62.7; 60.1; 4	52.0-67.9; 60.0; 12	16.6-22.5; 19.4; 9	15.6-19.8; 17.6; 12	68.7-87.9; 79.4; 7	67.6-89.2; 74.0; 10	61.3-79.7; 66.8; 12
TM: *Ny*. cf. *khinzir*		21.9	21.4	69.5	67.9	64.4					
TM: *Ny*. cf. *australis*		25.2-28.0; 26.6; 2	23.9-26.3; 25.1; 2	70.0-83.1; 76.5; 2	66.4-78; 72.2; 2	64.4-72.7; 68.6; 2		25.7		92.4	85.6
Ny. *tulotos*	all	17.6-22.0; 19.8; 9	16.3-21.8; 19.0; 9	59.9-71.2; 66.3; 9	55.6-71.2; 62.1; 9	46.0-64.1; 55.8; 14	19.2-21.2; 20.5; 3	17.4-19.2; 18.2; 6	85.4-91.4; 88.8; 3	70.4-83.2; 78.7; 4	56.2-78.0; 66.2; 7
	LN					46.0	19.2	17.6-18.1; 17.8; 3	89.7	78.4-83.2; 80.8; 2	56.2-66.7; 62.9; 3
	UN	18.8-22.0; 20.4; 3	19.0-20.1; 19.5; 3	59.9-70.5; 66.1; 3	55.6-62.4; 60.1; 3	51.2-60.3; 55.9; 5					64.1
	AA	17.6-21.8; 19.5; 6	16.3-21.8; 18.9; 5	63.5-71.2; 66.4; 6	58.8-71.2; 63.2; 5	50.0-64.1; 56.8; 6	21.0	19.1-19.2; 19.2; 2	91.4	82.8	70.5-78.0; 74.2; 2
Ny. *australis*	all	20.0-24.7; 22.7; 4	19.2-27.7; 22.9; 7	67.6-79.5; 75.0; 4	64.2-84.0; 72.1; 7	57.5-77.4; 66.1; 10	23-28.2; 25.2; 8	20.1-24.4; 22.4; 12	79.3-98.0; 89.7; 8	77.7-91.9; 84.1; 8	63.6-84.8; 76.4; 12
	LW		23.5-27.7; 25.6; 2		64.9-73.3; 69.1; 2	61.4-69.3; 65.4; 2	23.0-28.2; 25.5; 5	21.8-24.4; 23.6; 5	79.3-94.8; 89.2; 5	78.5-90.7; 85.3; 4	63.6-84.8; 75.1; 6
	AA	20.0-23.6; 22.1; 3	22.4-22.5; 22.5; 2	67.6-79.5; 75.2; 3	75.4-78.1; 76.8; 2	64.6-77.4; 68; 4	23.4-24.1; 23.8; 2	20.1-22.6; 21.3; 4	83.0-98.0; 90.5; 2	77.7-91.9; 83.5; 3	80.2-83.3; 81.8; 2
Ny. *kanamensis*		26.3	22.0	78.5	65.7	56.7-74.7; 65.7; 2	23.0	23.8	102.2		88.8
Ny. *devauxi*		17.7-18; 17.9; 2	15.2-16.7; 16.0; 3	73.2	62.0-65.0; 63.5; 2	44.4-54.7; 49; 3	16.0-19.0; 17.5; 2	15.4-17.9; 16.6; 4	76.2-88.0; 82.1; 2	72.7-77.7; 75.9; 3	66.8
Ny. *waylandi*		15.2		66.7			15.5-16.0; 15.8; 2		72.1-78; 75.1; 2		73.5
Ny. *kuseralensis*		19.8	18.7	71.7	67.8	50.7					

Abbreviations: LN, Lower Nawata; UN, Upper Nawata; AA, Adu-Asa; LW, Langebaanweg; h1, maximal height of the first pair of cusps/-ids; h2, maximal height of the second pair of cusps/-ids; H1, hypsodonty index (100*(h1/w)) of the first pair of cusps/-ids; H2, hypsodonty index (100*(h2/w)) of the second pair of cusps/-ids; H-tal, hypsodonty index (100*(h-tal/w)) of the talon/-id.
