# Supplementary material for: A New Species of Nyanzachoerus (Cetartiodactyla: Suidae) from the Late Miocene Toros-Ménalla, Chad, Central Africa
Source: PLoS One. 2014 Aug 27;9(8):e103221. doi: 10.1371/journal.pone.0103221 (PMC4146473; doi:10.1371/journal.pone.0103221)
Supplement: Table S6 — Stable isotope data (δ13C and δ18O in ‰) obtained from carbonates of enamel apatite of Nyanzachoerus at TM 266 and TM 267. Abbreviations: *, specimens attributed to Ny. khinzir nov. sp. (other specimens are attributed to Nyanzachoerus sp.). (PDF) [file pone.0103221.s008.pdf]

Table S6. Stable isotope data ($\delta^{13}C$ and $\delta^{18}O$ in ‰) obtained from carbonates of enamel apatite of *Nyanzachoerus* at TM 266 and TM 267.

Specimens	$\delta^{13}C$	$\delta^{18}O$	Specimens	$\delta^{13}C$	$\delta^{18}O$
TM 266-01-172*	-9.0	-5.5	TM 266-03-S8	-5.9	-2.3
TM 266-01-173*	-10.0	-5.9	TM 266-us1	-6.2	
TM 266-01-223*	-6.6	-6.3	TM 266-us2	-9.5	-4.5
TM 266-01-280*	-2.9	-4.3	TM 266-us3	-7.6	-2.6
TM 266-01-386*	-1.5	-6.7	TM 266-us4	-9.3	-3.6
TM 266-01-420*	-9.7	-5.3	TM 267-01-002*	-10.1	-1.1
TM 266-01-438*	-7.6	-5.6	TM 267-01-011	-4.2	-2.4
TM 266-02-163	-5.6	-0.8	TM 267-01-014*	-6.4	-2.1
TM 266-02-170	-6.4	0.5	TM 267-01-015*	-7.6	-7.1
TM 266-02-184*	-8.6	-6.5	TM 267-01-016	-9.6	-1.2
TM 266-02-198*	-5.8	-6.2	TM 267-01-017	-6.8	-1.5
TM 266-02-213	-6.7	-9.0	TM 267-01-097*	-9.3	-2.6
TM 266-03-S2	-8.4	-1.2	TM 267-01-145	-10.3	-0.3
TM 266-03-S3	-6.2	-5.6	TM 267-02-046	-6.6	-1.3

Abbreviations: *, specimens attributed to *Ny. khinzir* nov. sp.; other specimens are attributed to *Nyanzachoerus* sp.
