# Supplementary material for: A New Species of Nyanzachoerus (Cetartiodactyla: Suidae) from the Late Miocene Toros-Ménalla, Chad, Central Africa
Source: PLoS One. 2014 Aug 27;9(8):e103221. doi: 10.1371/journal.pone.0103221 (PMC4146473; doi:10.1371/journal.pone.0103221)
Supplement: Text S1 — Specimens of Nyanzachoerus from Toros-Ménalla, Chad. (PDF) [file pone.0103221.s010.pdf]

Text S1. Specimens of *Nyanzachoerus* from Toros-Ménalla, Chad.

Nyanzachoerus khinzir nov. sp.

Referred specimens. TM 5-01-001, right corpus with M/3; **TM 9-01-005**, fragmentary, edentulous rostrum; **TM 9-01-011**, juvenile mandible with left I/2, P/3-M/2 and right C/1, P/4-M/2; **TM 9-01-013**, palate fragments; **TM 9-01-017**, left maxilla with P3/-M3/; **TM 9-01-018**, right corpus, edentulous; **TM 9-01-020**, right corpus with M/1-M/3; **TM 9-01-021**, left corpus fragment with M/3; **TM 9-01-024**, right corpus with P/3 and roots of P/2, P/4; **TM 9-01-037**, left corpus with P/2-M/2; **TM 9-01-038**, left hemimandible with P/3-M/3; **TM 9-01-039**, right maxilla with P3/-M3/; **TM 9-01-040**, fragmentary left corpus with M/2, M/3 fragment; **TM 9-01-041**, left M/3; **TM 9-01-042**, right corpus fragment with M/3; **TM 9-01-043**, maxilla and corpus fragments, with left M3/?; **TM 9-01-046**, right M3/; **TM 9-01-047**, various tooth remains, including left M/1, M/3; **TM 9-01-048**, fragmentary maxillae with left P4-M2/ and right M1/-M3/; **TM 9-01-347**, M/2 or M/3; **TM 9-01-348**, left M3/; **TM 9-01-411**, left fragmentary corpus with M/3; **TM 9-01-420**, fragmentary left corpus with dP/4-M/1; **TM 9-01-421**, right M2/; **TM 9-01-527**, upper teeth, including left P3/, P4/, M2/ and right M3/; **TM 9-01-528**, fragmentary palate with right P4/-M3/ and left P3/-M3/; **TM 9-02-006**, left mandibular symphysis fragment; **TM 9-02-008**, left M/3; **TM 9-05-001**, rigth corpus with P/3-M/3; **TM 9-05-002**, left corpus with M/1-M/2; **TM 14-98-001**, partial cranium; **TM 17-97-001**, partial cranium with right P4/-M3/ and left P3/-M3/; **TM 23-97-001**, mandibular fragments, including C/1, P/2, P/3-M/1; **TM 38-97-001**, fragmentary palate with broken P/3-M/3; **TM 39-97-002**, partial rostrum with left P1/-P2/ and right P2/; **TM 39-97-003**, fragmentary left corpus with M/3; **TM 39-99-004**, right corpus with P/3-M/3; **TM 43-00-002**, palate with right and left P3/-M3/; **TM 43-01-010**, right corpus with P/4-M/2; **TM 45-99-012**, male mandibular symphysis; **TM 53-98-002**, palate with left P4/-M3/ and right P3/-M3/; **TM 53-98-013**, partial cranium with right P2/-M1/ and left P3/-M2/; **TM 54-00-003**, fragmentary right corpus with eroded M/3; **TM 55-98-012**, fragmentary left corpus with M/3; **TM 60-99-009a**, fragmentary left corpus with M/3 plus ; **TM 60-99-009b**, right corpus with P/4-M/2; **TM 60-99-009c**, left M/2; **TM 60-99-009d**, left P/4; **TM 60-99-015**, right maxilla with P3/-M3/; **TM 61-98-006**, fragmentary left corpus with M/1 and unerupted M/2; **TM 62-01-001**, molar; **TM 66-98-001**, fragmentary right corpus with P/3-M/1; **TM 66-98-002**, fragmentary right corpus with P/3-M/3 plus fragmentary left corpus with P/4-M/3; **TM 74-99-007**, left M/3 talonid; **TM 74-99-017**, fragmentary right corpus with P/3-P/4; **TM 75-99-001**, upper cheek teeth including right P3, P4/, M2/, M3/; **TM 76-00-003**, left corpus fragment with M/3;

TM 76-01-001, mandibular symphysis; **TM 76-06-032**, dentition including right and left M3/; **TM 76-06-033**, upper cheek teeth; **TM 78-99-002**, right and left corpora with P/3-M/3; **TM 78-99-005**, left corpus with M/1 roots and M/2-M/3; **TM 79-99-001**, eroded mandibular symphysis and left corpus with M/2-M/3; **TM 79-99-002**, eroded, edentuous mandible; **TM 79-99-003**, eroded mandible with left P/4-M/3 and roots of right P/3-M/1, and various fragments; **TM 80-99-002**, fragmentary left corpus with dP/4-M/1; **TM 82-01-001**, left maxilla with P3/-M2/ and eroded M3/; **TM 82-01-002**, right maxilla with P3/-M3/; **TM 90-00-001a**, cranial fragment plus eroded left corpus with broken M/2-M/3; **TM 90-00-001b**, right corpus with P/3-M/2 roots and fragmentary M/3; **TM 90-01-091**, left M3/ talon; **TM 90-03-031**, right corpus with P/4-M/2; **TM 90-06-040**, fragmentary cranium with right M3/; **TM 90-99-046**, partial cranium; **TM 92-01-038**, palate with right and lef P3/-M3/ plus right and left eroded corpora with P/3-M/3 and P/1-M/3; **TM 92-01-039**, maxillae with right and left P3/-M3/; **TM 92-06-011**, M/2; **TM 92-99-016**, left M3/; **TM 94-99-002**, left maxilla with P4/-M2/; **TM 98-01-003**, right P/3; **TM 100-00-002**, fragmentary right maxilla with M3/; **TM 100-00-011**, fragmentary rith corpus with P/4-M/3; **TM 100-00-013**, subcomplete mandible with left and right I/1, P/2-M/3 and right C/1; **TM 101-00-001**, left corpus with M/1-M/3; **TM 101-06-007**, left corpus with roots of P/2-M/3; **TM 101-06-008**, left and right hemimandibles with roots of P/4-M/3 and M/1-M/3; **TM 101-06-009**, juvenile rigth and left corpora with dP/2-M/1 and roots of dP/2-M/1; **TM 103-00-007**, palate with left and right P3/-M3/; **TM 112-00-057**, teeth, including two eroded upper P and eroded left and right M3/ plus fragmentary M/3; **TM 112-00-059**, lower incisors plus right and left corpora with P3/-M3/; **TM 112-00-118**, left maxilla with P3/-M3/; **TM 112-00-126**, eroded left corpus with M/2-M/3; **TM 112-00-153**, fragmentary right M/3; **TM 112-00-165**, right M/3; **TM 115-06-057**, maxillae with left P3/-M3/ and right P4/-M3/; **TM 115-10-005**, fragmentary right corpus with dP/4-M/1; **TM 116-00-003**, right corpus with eroded M/1-M/3; **TM 120-01-005**, right maxilla fragments with P3/-M2/; **TM 124-01-003**, nuchal portion of braincase; **TM 130-01-001**, mandible with rigth P/2-M/3 and left P/3-M/3, and other mandibular and postrcranial remains; **TM 140-06-001**, left and right corpora with M/3 and M/2-M/3; **TM 146-01-001**, left corpus with dp/2-dP/4 and M/1 alveolus; **TM 146-01-020**, maxillae with right and left P4/-M3/ + fragments; **TM 152-01-001**, right maxilla with eroded P2/-M3/; **TM 153-01-011**, fragmentary right corpus with M/1; **TM 154-01-001**, right M3/; **TM 154-01-002**, right M3/; **TM 161-01-001**, M/3 talonid; **TM 168-01-001**, left fragmentary corpus with M/3 fragment; **TM 169-05-001a**, left corpus with P/3-M/3; **TM 169-05-001b**, right corpus with P/3-M/3; **TM 170-01-013**, fragmentary left corpus with P/3-M/2; **TM 170-01-015**, eroded left maxilla

with P4/-M3/; **TM 170-01-019**, fragmentary mandible with symphysis with right left C/1 and right P/3, plus fragmentary left corpus with P/3-M/2, plus fragmentary right corpus with P/4-M/1, plus right C/1; **TM 170-01-020**, right and left corpora with P/3-M/3; **TM 170-01-023**, left M/3; **TM 171-01-024**, right maxilla with P3/-P4/; **TM 171-01-025**, right fragmentary corpus with eroded M/3; **TM 171-01-031**, fragmentary mandible with right and left canines, right P/3-P/4, M/2-M/3 and left P/4-M/3; **TM 171-01-032**, juvenile fragmentary palate with left dP4/-M1/ and right P4/ germ plus partial left corpus with dP/4-M/2; **TM 172-01-005**, right and left corpora with C/1 fragments, right P3/-M3/, left P/2-M/3; **TM 172-01-009**, right and left corpora with P3/-M3/ plus symphysis; **TM 172-01-011**, fragmentary left maxilla with P3/-M3/ plus right P3/ fragment, M2/ and fragmentary M3/; **TM 172-01-013**, eroded left maxilla with P3/-M3/; **TM 172-01-014**, left corpus with P/3-M/3 plus two C/1 fragments; **TM 172-01-020**, left P/2; **TM 172-01-053**, left maxilla with P3/-M3/; **TM 172-01-064**, left P/3-P/4; **TM 172-01-066**, fragmentary left maxilla with P3/-M1/ plus left M3/ plus right M3/ talon plus fragments; **TM 175-09-036c**, fragmentary right corpus with M/3 talonid; **TM 175-09-036e**, fragmentary left corpus with P3/-P4/; **TM 176-01-011**, fragmentary left hemimandible and teeth; **TM 176-01-021**, left M/2; **TM 176-01-027**, right M/3 talonid; **TM 178-01-024**, left M/3; **TM 178-01-032**, lower dentition including left and right M/3; **TM 178-02-001**, corpus fragments with left P/3-P4 and right P/3; **TM 180-01-071**, left M/3; **TM 183-01-002**, palate with left and right P3/-M3/ and braincase fragments; **TM 186-01-001a**, left corpus with P/3-M/3; **TM 186-01-001b**, right corpus with P/2-M/3; **TM 186-01-001c**, eroded mandibular symphysis with left C/1; **TM 187-01-001**, fragmentary left corpus with P/3-M/1 roots and M/2-M/3 fragments; **TM 195-01-021**, mandibular symphysis; **TM 195-01-022**, left corpus with P/4-M/3; **TM 199-01-003**, left M3/ germ; **TM 202-01-001**, fragmentary dentition, including left P2/ and M3/, left M/2; **TM 202-05-002**, right hemimandible with C/1, P/4-M/3; **TM 204-01-007**, fragmentary corpus with eroded M/1-M/2; **TM 205-01-010**, right M/2 and other tooth fragments; **TM 205-09-001a**, right maxilla with P3/-M3/; **TM 205-09-001b**, left maxilla with P3/-P4/; **TM 214-01-007**, mandibular symphysis and edentulous right corpus; **TM 215-01-053**, left maxilla with M2/-M3/; **TM 215-01-055**, M/3 fragment; **TM 215-01-058**, fragmentary left corpus with P/4-M/2 and M/3 fragment; **TM 215-01-094**, left maxilla with P3/-M3/; **TM 215-01-095**, left corpus with P/3 alveolus and P/4-M/2; **TM 215-01-109**, left P3/-P4/; **TM 215-01-110**, fragmentary right maxilla with dP2/-dP4/; **TM 215-01-116**, right M/3; **TM 215-01-119**, eroded left maxilla with P3/-M3/; **TM 215-10-001**, left M3/ germ; **TM 222-01-003**, right corpus with P/2 alveolus and P/3-M/3; **TM 225-01-001a**, right portion of cranium; **TM 225-01-001b**, craniomandibular fragments including dentition (M3/,

P/3-M/3) and other fragments; **TM 226-01-037**, left M2/ and M3/; **TM 233-08-001**, complete mandible with left P/3-M/3 and right P/2-M/3; **TM 242-01-024**, right corpus with M/2-M/3; **TM 242-02-005**, right M3/; **TM 242-02-030**, right M/3; **TM 242-06-007**, left corpus with M/2-M/3; **TM 242-06-008**, left corpus with P/3-M/2; **TM 244-01-002**, left M/3; **TM 244-04-001**, left M/3; **TM 246-01-001**, juvenile mandible with left and right P/4-M/2, unerupted M/3s; **TM 246-01-002**, left corpus fragment with M/2-M/3; **TM 246-01-003**, left palate fragment with I3/ and P2/-P3/ ; **TM 246-01-004**, right palate fragment with I1/-C1/ alveoli and P1/-P2/; **TM 254-01-001**, right corpus with P/4-M/3; **TM 254-01-002**, right M/3; **TM 254-01-003**, right M/2; **TM 254-04-003**, right M/3; **TM 255-01-004**, right M3/; **TM 256-06-005**, eroded mandibular symphysis with roots of left I/1-P/3 and of right I/1-P/1; **TM 258-01-001**, juvenile palate with left and rigth P4/-M2/; **TM 258-10-008**, right M3/ talon; **TM 259-01-036**, left M3/; **TM 259-02-017**, upper dentition, including upper left M3/; **TM 263-01-018**, right M/3 and premolar fragment; **TM 265-01-008**, left maxilla with eroded M2/-M3/; **TM 266-01-020**, fragmentary palate with left M2/-M3/; **TM 266-01-172**, M2/; **TM 266-01-173**, right M/3; **TM 266-01-223**, right M/2; **TM 266-01-280**, right corpus with M/1-M/2; **TM 266-01-289**, left corpus with eorded dP/3-M/1; **TM 266-01-386**, left M/3 germ; **TM 266-01-420**, P3/; **TM 266-01-438**, right P3/; **TM 266-02-184**, left corpus with eroded P/3-M1; **TM 266-02-198**, right M/3; **TM 266-03-091**, right M2/ germ; **TM 266-03-293**, palate with right P2/-M3/ and left P3/-M2/; **TM 266-03-308**, left M/2 germ; **TM 266-05-067**, right M3/; **TM 266-09-009**, right M3/ germ; **TM 266-11-028**, palate fragments with right P2/-M1/, M3/ and left P3/-P4/, M3/, plus partial mandible with right and left P/2-M/3; **TM 266-11-033**, fragmentary, distorted mandible with left P/2-M/2, eroded M/3 and right P/1-P/3; **TM 267-01-002**, right corpus with eroded P/2-M/3; **TM 267-01-008**, left M3/ germ; **TM 267-01-014**, right P/4; **TM 267-01-015**, left M/1; **TM 267-01-097**, left P3/; **TM 267-02-049**, eroded P/3, P4/; **TM 271-06-024**, left M1/; **TM 271-06-025**, right M/2-M/3; **TM 271-06-026**, left corpus with P/3-M/3; **TM 274-02-011**, left maxilla fragment with C/1 root and P/1 alveolus; **TM 274-10-001a**, distorted right M3/; **TM 274-10-001b**, distorted right M2/; **TM 274-10-001c**, distorted right P4/M1/; **TM 275-06-003**, left maxilla with eroded P3/-M2/; **TM 275-06-062**, right M3/; **TM 275-06-063**, left M3/; **TM 276-01-003**, left corpus with P/2-M/3; **TM 276-02-010**, juvenile jaws with dP2/-dP4/-M1/ and dP/2-dP/4-M/1; **TM 276-03-006**, partial juvenile cranium with right and left dP/2-M1/; **TM 276-03-031**, right M/3 germ and M fragment; **TM 276-08-005**, left corpus with dP/2-M/1; **TM 278-09-017**, right P/3-P/4; **TM 278-09-018**, left M/3; **TM 279-02-009**, right M2/; **TM 279-06-030**, juvenile left maxilla with dP2/-M1/; **TM 279-06-037**, mandibular symphysis; **TM 282-02-010**, left M/1; **TM 282-02-011**, left M/3;

TM 283-01-010, left maxilla with P4/-M2/; **TM 284-01-001**, right corpus with M2/ and M3/ alveolus; **TM 289-01-005**, left M/3; **TM 289-01-007**, fragmentary right M3/; **TM 289-02-041**, M2/; **TM 291-01-001a**, right corpus with P/4-M/3; **TM 291-01-001b**, left corpus with M/1-M/3; **TM 292-02-018**, left corpus with P/2 alveolus and P/3-P/4; **TM 292-09-002**, left fragmentary corpus with M/2-M/3; **TM 297-01-019**, left M/1; **TM 298-01-001**, right maxilla with P3/-M2/; **TM 299-02-004**, right M3/; **TM 301-02-002**, fragmentarty left corpus with M/1-M/2; **TM 301-02-022**, juvenile right corpus with dP/4-M/2; **TM 305-02-008**, juvenile right corpus with dP/4-M/2; **TM 305-02-011**, right maxilla with P3/-M3/; **TM 308-01-001**, partial cranium; **TM 309-01-010**, M/2; **TM 309-01-013**, left M/2; **TM 317-03-001**, fragmentary premaxillae and maxillae, including rigth P3/-M3/; **TM 321-01-002**, left corpus fragment with M/3; **TM 327-04-009**, fragmentary right M/3; **TM 328-01-001a**, left corpus with P/2-M/3; **TM 328-01-001b**, left corpus with M/1-M/2; **TM 331-05-001**, right corpus with P/4-M/3; **TM 335-05-007**, right M/3; **TM 337-02-002**, upper and lower dentition including incisors, canines, right M3/, left P/3-M/3, right P/2-M/3; **TM 339-02-003**, fragmentary right corpus with P/4-M/3; **TM 339-02-004**, right M3/; **TM 339-02-005**, left maxilla with M1/-M2/; **TM 340-02-001a**, cranial fragment with right M1/-M2/; **TM 340-02-001b**, right corpus with C/1, alv. P/1, P/3-M/3; **TM 341-05-007**, partial cranium with right P2/-M3/ and left P3/-M3/; **TM 347-02-002**, right M/2; **TM 360-02-023**, left corpus with P/3-P4; **TM 360-02-031**, right corpus fragment with M/3; **TM 360-02-036**, left M/3; **TM 361-02-036**, left M/2 germ and various teeth; **TM 372-02-001**, left M/3; **TM 377-04-005**, juvenile right corpus with dP/2-M/1; **TM 379-04-003**, various mandible fragments and dentition, including right P/3-P/4 and left M/1-M/3; **TM 382-05-012**, juvenile right corpus with dP/4-M/1.

Nyanzachoerus cf. *khinzir*

Referred specimens. TM 9-01-019, left maxilla with P2/, P4/-M3/.

Nyanzachoerus* cf. *australis

Referred specimens. TM 29-97-002, eroded right corpus with P/2-M/3; **TM 70-99-001**, tooth fragment, including fragmentary M/3; **TM 71-99-001**, left corpus with P/3-M/3; **TM 72-01-001**, eroded right M1/-M2/, M3/ plus left corpus with P/3-M/2 and M/3 partially encrypted; **TM 72-01-002**, left hemimandible with eroded M/2-M/3; **TM 73-01-005**, right corpus with broken P/3-M/3; **TM 193-01-002**, left M/3; **TM 230-01-001**, palate with right P3/-M3/ and left P4/-M3/ plus eroded right and left corpora with broken P/3-M/3 and P/4-

M/3; **TM 230-01-002**, juvenile left hemimandible with unerupted P/4 and M/2; **TM 230-01-004**, eroded right M/2; **TM 230-01-005**, partial mandible; **TM 230-01-006**, eroded palate with roots of left and right P3/-M3/; **TM 230-01-008**, eroded left corpus with M/2-M/3; **TM 230-01-009**, eroded rigth P/4-M/3 plus eroded left M3/.

Nyanzachoerus sp.

Referred specimens. TM 7-01-035, lower molar germ fragment; **TM 9-00-013**, palate fragment with roots of left and right M2/-M3/; **TM 9-01-044**, mandibular symphysis fragment with roots of left and right I/1, I/2, C/1 and right P/2; **TM 9-01-045**, right hemimandible with eroded P/4-M/3; **TM 30-97-002**, juvenile left hemimandible with roots of I/1-M/1; **TM 39-06-029**, fragmentary palate with eroded teeth; **TM 40-97-002**, crushed and eroded cranium with worn out and eroded teeth; **TM 55-01-002**, fragmentary jaw with roots; **TM 60-99-026**, eroded mandibular symphysis; **TM 74-01-021**, M/3 fragment; **TM 76-00-001**, fragmentary right corpus with M/3 germ; **TM 85-99-003**, eroded right and left hemimandibles with P/3-M/3 and P/4-M/3; **TM 87-02-002**, right hemimandible with M/1-M/2 alveoli and unerupted M/3; **TM 92-01-037**, C1/; **TM 92-01-048**, right maxilla with dP3/-dP4/; **TM 92-06-076**, eroded dental series; **TM 92-06-084**, left M/2; **TM 92-99-018**, fragmentary left corpus with P/2 roots and fragmentary P/3 and eroded M/2-M/3, plus right corpus with eroded M/2-M/3; **TM 92-99-026**, hemimandible fragment with eroded P/2-P/3; **TM 107-10-001**, fragmentary mandible with eroded teeth; **TM 112-00-036**, eroded right corpus with dP/4-M/1; **TM 112-00-040**, left upper canine; **TM 112-00-097**, eroded left P3/-P4/; **TM 112-00-135**, left M2/ germ; **TM 112-00-167**, left M2/ germ fragment; **TM 112-00-183**, fragmentary left maxilla with M3/ roots; **TM 112-00-186**, tooth fragment, possibly M3; **TM 112-00-189**, M fragment; **TM 112-00-207**, eroded M3; **TM 112-01-001**, right hemimandibular corpus with eroded M/1-M/3; **TM 115-00-128**, fragmentary hemimandible with roots; **TM 115-06-121**, eroded mandibular symphysis; **TM 119-08-025**, fragmentary right corpus with dP/4-M/1; **TM 120-04-003**, M fragment; **TM 128-01-001**, crushed maxilla with eroded left dP2/, dP4/-M2/ and rigth dP3/-M2/; **TM 132-01-009**, right hemimandible with roots of P/3-M/1; **TM 146-01-002**, left M/2 germ fragment; **TM 147-01-008**, upper dentition fragments, including left P2/ and P3/, right P4/ and M2/; **TM 154-01-004**, C1/; **TM 154-01-005**, M3 talon/id fragment; **TM 160-01-004**, cheek tooth fragments; **TM 161-01-001**, eroded P/4; **TM 172-01-061**, P fragment; **TM 172-01-067**, eroded lower molar; **TM 175-09-036 (pro parte)**, indeterminate cranial fragments; **TM 180-01-072**, left hemimandible fragment with eroded M/3; **TM 191-01-024**, fragmentary right corpus with M/1-M/3 roots; **TM 205-01-011**, fragmentary P4/,

M1/, and left and right M2/, all eroded; **TM 205-09-002**, right maxilla with eroded P4/-M2/; **TM 205-09-003**, eroded upper dentition; **TM 215-01-054**, eroded left P4/ and left and right M1/; **TM 215-05-016**, matrix covered dentition, including P3/, P4/, M2/, M3/; **TM 215-05-018**, distorted left M1/; **TM 215-06-025**, right maxilla with eroded P3/-M3/; **TM 215-09-016**, left maxilla with eroded dP2/-M1/; **TM 215-10-001 (pro parte)**, dental fragments; **TM 218-01-012**, left and right maxilla fragments; **TM 219-01-067**, fragmentary M3; **TM 219-01-077**, right hemimandible with eroded P/3-P/4; **TM 219-05-024**, M fragment; **TM 226-01-033**, eroded right P3/; **TM 227-01-006**, eroded right M/3; **TM 233-08-003**, fragmentary right maxilla with unerupted P4/, M1/ ; **TM 242-04-029**, fragmentary upper M; **TM 242-04-030**, rigth corpus fragment with eroded P/4 and M/3; **TM 242-04-055**, juvenile mandibular symphysis with left and rigth I/1; **TM 242-05-001**, rigth hemimandible with dP/3-dP/4 alveoli and eroded M/1; **TM 247-06-XXX**, M/2; **TM 252-05-008**, upper I; **TM 254-02-004**, C/1; **TM 254-04-038**, mesial fragment of upper M; **TM 257-04-007**, mesial fragment of lower M; **TM 258-04-001**, left lower I; **TM 266-01-021**, fragmentary M3; **TM 266-01-396**, left premaxilla fragement with I/1-I/3 alveoli; **TM 266-02-163**, molar; **TM 266-02-170**, jaw fragment with eroded tooth; **TM 266-02-213**, canine; **TM 266-03-064**, upper decidual dentition and unerupted teeth; **TM 266-03-276**, upper I; **TM 266-03-278**, distal part of M3 talon/-id; **TM 266-03-S2**, fragmentary tooth; **TM 266-03-S3**, fragmentary tooth; **TM 266-03-S8**, fragmentary tooth; **TM 266-04-006**, M fragment; **TM 266-05-056**, fragmentary upper dentition; **TM 266-06-010**, fragmentary dP/4 ?; **TM 266-us1**, fragmentary tooth; **TM 266-us2**, fragmentary tooth; **TM 266-us3**, fragmentary tooth; **TM 266-us4**, fragmentary tooth; **TM 267-01-011**, upper canine; **TM 267-01-010**, left C1/ male; **TM 267-01-016**, fragmentary M3 talon/-id; **TM 267-01-017**, right M/3 talonid fragment; **TM 267-01-145**, right corpus with eroded M/1-M/2; **TM 267-01-146**, left hemimandible with eroded M/2-M/3; **TM 267-02-046**, M3 fragment; **TM 267-03-013**, right hemimandible with eroded M/2-M/3; **TM 271-02-002**, eroded hemimandible with P/4-M/1; **TM 271-05-002**, right corpus with M/2-M/3; **TM 273-06-018**, left C1/; **TM 275-06-040**, eroded mandibular symphysis; **TM 276-01-001**, right C/1; **TM 276-02-009**, juvenile palate with left dP3/-M1/ and right dP3/-dP4/; **TM 276-02-049**, right maxilla with eroded P3/-M2/; **TM 276-03-005**, fragmentary juvenile left corpus with fragmentary M/1; **TM 276-03-039**, eroded tooth fragments; **TM 282-01-003**, right maxilla with C1/-P1/; **TM 289-01-008**, eroded M3; **TM 289-03-003**, unerupted M/2; **TM 293-01-020**, right C/1 (female?); **TM 299-05-006**, right M3/; **TM 299-05-010**, right lower I; **TM 301-01-002**, right hemimandible with eorded P/2-P/4; **TM 301-02-011**, M/3 talonid fragment; **TM 305-02-007**, canine; **TM 314-06-015**, left hemimandible with eroded M/2-M/3; **TM 317-03-**

003, fragmentary mandibular symphysis; **TM 325-01-003**, right C1/ male; **TM 360-02-018**, eroded, fragmentary mandibular symphysis; **TM 380-04-004**, left maxilla with eroded P4/-M2/; **TM 382-05-014**, left upper I.
