# Supplementary material for: A New Species of Nyanzachoerus (Cetartiodactyla: Suidae) from the Late Miocene Toros-Ménalla, Chad, Central Africa
Source: PLoS One. 2014 Aug 27;9(8):e103221. doi: 10.1371/journal.pone.0103221 (PMC4146473; doi:10.1371/journal.pone.0103221)
Supplement: Text S2 — Summary of the dental nomenclature. (PDF) [file pone.0103221.s011.pdf]

Text S2. Dental nomenclature, from Boisserie et al. 2008, Zool. J. Linn. Soc. [1].

The complexity scores (CS) for upper and lower third molars are defined in the list below and illustrated in Fig. S1. Dental nomenclature follows [1].

The nomenclature used here (see also Fig. S1) was built on classical dental serial homologies. The work of Hershkovitz [2] was a major source for defining homologous structures and naming them, but other sources were also considered [3-7]. These works emphasized dental structures that are expressed as relief on tooth crowns. Structures expressed as depressions, in particular groove systems, were also demonstrated to be important features of tooth morphology (see notably Hünermann's and Orliac's works on suoids [8-10]). Grooves are often more resistant to wear than salient structures, and the presence of grooves is not dependent on that of crests (and vice versa). Therefore, both systems (relief- and depression-based) are viewed here as fully complementary and were integrated in the proposed nomenclature. On this ground, two steps were necessary to establish a congruent terminology: 1) naming and defining the common structures appearing on all tooth crowns; 2) naming and defining particular cusps/-ids and their elements. Tooth orientation terminology used in this work (Fig. S1) follows recommendations formulated by Smith & Dodson [11].

Definitions

Terminology for basic structures

Ambiguity appears at a first level in dental nomenclature: that of basic crown structures. Cusps/-ids and cingula/-ids have a universal meaning and do not need particular definitions, but this is not the case for other structures including accessory cusps/-ids, crests, lobes, grooves, valleys, and basins. They have been used differently for different groups, and there was a need to adopt clear definitions.

Style/-id. A marked eminence of the cingulum/-id, sometimes developed as a distinct 'cusplet' (for exaggerated development, see parastyles of the anthracotheriid *Brachyodus*). Fig. S1 illustrates the usual positions and names of styles/-ids.

Cristyles/-ids. Elongated invaginations of the cingula/-ids extending in the transverse valley (ectocristyle/-id on labial side, ectocristyle/-id on lingual side; Fig. S1).

Valleys. On molars, the sagittal valley separates lingual and labial cusps/-ids, whereas the transverse valley separates mesial and distal cusps/-ids. When they are particularly wide, they can be called “basins”.

Crista/-id. An elongated structure of approximate apico-basal direction on cusps/ids. When unworn, cristae/-ids can be marked by a salient ridge (*e.g.*, in Hippopotaminae) or smooth with no ridge at all, (*e.g.*, in Suidae). Smooth cristae/-ids are often termed as ‘lobes’, a name which is avoided here, as it is often not possible to differentiate a salient ridge from a lobe on worn teeth. Cristae/-ids that appear on cusps/-ids but that are not identifiable to a major crista, are particularly reduced and/or not directed toward the apex are referred to as “accessory cristae/-ids”.

Conule/-id. A cusp/-id secondarily developed on a crista/-id.

Fossa/-id. An elongated groove of approximate apico-basal direction on cusps/-ids. Fossae/-ids can be wide or narrow, deep or shallow (*e.g.*, wide and deep in hippopotamids; narrow and deep in suids; shallow and narrow in paleochoeres). Fossae/-ids can emphasize smooth cristae/-ids. The fossae/-ids that partially isolate a conule/-id from its cusp/-id are called accessory fossae/-ids (they are not directed toward the cusp/-id apex). When these accessory fossae/-ids coalesce, completely isolating the conule/-id, the resulting fossa/-id is termed “transverse fossa/-id”.

Remark 1. A crista/-id can be identified without fossa/-id if it: (1) is marked by a salient ridge; (2) constitutes a clear elongation of the cusp (but in that case, wide and shallow fossae/-ids often accompany the elongation). Similarly, a fossa/id can appear without clear association to a salient ridge or to a distinct cusp elongation – this is notably seen in suoids. For these reasons, both networks of fossae/-ids and cristae/-ids need to be considered when describing teeth.

Remark 2. Salient ridges can appear on the side of fossae/-ids, especially on upper molars (*e.g.*, lingually to a postprotofossa, see protocone on Fig. I of online supplementary material) without being central to a ‘lobe’. These ridges, sometimes termed as ‘ribs’, if salient, are considered as cristae/ids not immediately followed by another fossa/id.

Definitions for molar cusps/-ids and their structures

Cusp/-id nomenclature used here (see Fig. S1) mainly follows the Cope-Osbornian theory for homology of mammalian teeth as reviewed and revised by Hershkovitz [2]. In the families studied here, the four main molar cusps/-ids are: paracone, protocone, metacone, and metaconule on upper molars (there is no true hypocone, which is defined as a cusp originating

from the cingulum and not part of the trigon); protoconid, metaconid, hypoconid, and entoconid on lower molars. The fifth distal cusp appearing on M^3 received various names (*e.g.*, ‘distal median cusp’, ‘talon’, ‘terminal pillar’, ‘pentacone’). It originates from the cingulum and is most likely homologous to the distostyle [2]. When it is strongly developed, as in many suids, we propose to name it “distocone”. A similar cuspid on M_3, present in all studied families, is also of cingular origin and homologous to the distostylid, but the name hypoconulid is preferred to distoconid because it has been extensively used in literature.

The proposed definitions of cusp/-id primary structures were first based on homology as indicated by Hershkovitz [2]. Homologies were tentatively identified by using the principle of connexion and historical criteria for features with well-known evolution within families. They principally concern pre- and postcristae/-ids (and their conules/-ids), styles/stylids, and cingulum/-ids (see Fig. S1). Because they may be applied to structures of quite different shape and relations between different groups (especially when not all structures are present or recognizable, such as reduced styles/stylids), homologies have sometimes been difficult to recognize. In these cases, most parsimonious interpretations were systematically favoured.

Additional structures, often more variable and considered as secondarily evolved, were identified following their position relatively to the primary structures. These secondary structures are not a priori smaller and/or less extended than primary ones. The homology of secondary structures is essentially based on topographic and parsimony criteria. Figure 2 illustrates primary structures for molars; Figs. I and II of online supplementary material illustrates primary and secondary structures for molars.

Preprotocrista. Crista of the protocone that joins the mesiostyle on the mesial cingulum. The mesiostyle can be absent or indistinguishable from the parastyle, so the junction to the mesial cingulum remains the principal criterion to define the preprotocrista.

Postprotocrista. Crista of the protocone that reaches the center of the tooth (disto-labial orientation) and often extends towards the premetacristule.

Paraconule. Conule developed on the preprotocrista. This term is preferred to that of ‘preprotoconule’ because of its generalized use in literature. The paraconule may develop its own network of cristae and fossae: the preparacristule joins the cingulum; the posparacristule is directed medially; the ectoparacristule joins the protocone (preprotocrista).

Premetacristule. Crista of the metaconule that reaches the center of the tooth (mesio-labial orientation) and often extends towards the postprotocrista.

Postmetacristule. Crista of the metaconule that joins the distostyle of the distal cingulum. The distostyle can be absent or indistinguishable from the metastyle, so the junction to the distal cingulum remains the principal criterion to define the postmetacristule.

Preparacrista. Crista of the paracone that joins the parastyle on the mesial cingulum (see preprotocrista for comments).

Postparacrista. Crista of the paracone that joins the premetacrista and may extends toward the ectostyle (mesostyle).

Premetacrista. Crista of the metacone that joins the postparacrista and may extends toward the ectostyle (mesostyle). The conule sometimes developed on the premetacrista is called centroconule in order to avoid confusion with that of the premetacristule.

Postmetacrista. Crista of the metacone that joins the metastyle (see postmetacristule for comments).

Preprotocristid. Cristid of the protoconid that is directed lingually and parallel to the mesial cingulid. It can joins the cingulid or the premetacristid.

Postprotocristid. Cristid of the protoconid that is directed disto-lingually and joins the postmetacristid.

Premetacristid. Cristid of the metaconid directed toward the lingual extremity of the mesial cingulid. It can extend labially to join the preprotocristid.

Endometacristid. Cristid of the metaconid directed labially toward the preprotocristid. The endometacristid generally joins the preprotocristid on its lingual wall.

Postmetacristid. Cristid of the metaconid that is directed disto-labially and joins the postprotocristid.

Preentocristid. Cristid of the entoconid that is directed mesio-labially and joins the lingual wall of the prehypocristid or the endohypocristid.

Postentocristid. Cristid of the entoconid that is directed disto-labially and joins the posthypocristid on its lingual wall or in convergence toward the distostylid.

Prehypocristid. Cristid of the hypoconid that is directed mesio-lingually and generally joining the postmeta- and/or postprotocristids. A conulid may develop on this cristid. It is named mesoconulid instead of prehypoconulid, the latter being used for the M_3 prehypocristulid (=distoconid) only.

Posthypocristid. Cristid of the hypoconid that joins the distostylid (the prehypocristulid on M_3s).

Prehypocristulid. Cristid of the M_3 hypoconulid that joins the posthypocristid. A prehypoconulid may develop on the prehypocristulid.

Posthypocristulid. Cristid of the M_3 hypoconulid that joins the lingual cingulid and, if present, the postentostylids.

Ectohypocristulid. Cristid of the M_3 hypoconulid that develops on the lingual wall. It can join the apex or the prehypocristulid lingually, and probably originates labially from the postectostylids.

Ectocristae/-ids. Cristae/-ids lateral to the precrista/-id and positioned in the mesial half of the cusp/-id.

Postectocristae/-ids. Cristae/-ids lateral to the postcrista/-id and positioned in the distal half of the cusp/-id.

Endocristae/-ids. Cristae/-ids on the median wall, between the precrista/-id and the postcrista/-id.

Fossae/-ids. They adopt the name of the crista/-id placed immediately mesio-medially to it.

Definitions for premolar cusps/-ids and their structures

The same cusp/-id terminology was used for premolars. However, it must be stressed that premolar and molar cusps/-ids bearing the same name are not necessarily homologous. Terms used to designate premolar cusps/-ids are here essentially attributed on the base of general position on the tooth (e.g., the “protocone” on P^3). Principal identifiable structures are illustrated on Fig. S1.

References

1. Boisserie J-R, Lihoreau F, Orliac M, Fisher RE, Weston EM, et al. (2010) Morphology and phylogenetic relationships of the earliest known hippopotamids (Cetartiodactyla, Hippopotamidae, Kenyapotaminae). Zoological Journal of the Linnean Society 158: 325–366.
2. Hershkovitz P (1971) Basic crown patterns and cusp homologies of mammalian teeth. In: Dahlberg AA, editor editors. Dental morphology and evolution. Chicago: The University of Chicago Press. pp. 95–150.
3. Butler PM (1978) Molar cusp nomenclature and homology. In: Butler PM,Joysey KA, editors. Development, Function and Evolution of Teeth. London: Academic Press. pp. 439–453.

4. Gentry AW,Hooker JJ (1988) The phylogeny of the Artiodactyla. In: Benton MJ, editor editors. The phylogeny and classification of the Tetrapods, Volume 2 : Mammals. Oxford: Clarendon Press. pp. 235–272.
5. Made J van der (1996) Listriodontinae (Suidae, Mammalia), their evolution, systematics and distribution in time. Contributions to Tertiary and Quaternary Geology 33: 3–254.
6. Tassy P (1996) Dental homologies and nomenclature in the Proboscidea. In: Shoshani J,Tassy P, editors. The Proboscidea Evolution and Palaeoecology of Elephants and their Relatives. Oxford: Oxford University Press. pp. 21–25.
7. Van Valen L (1966) Deltatheridia, a new order of mammals. Bulletin of the American Museum of Natural History 132: 1–128.
8. Hünermann KA (1968) Die Suidae (Mammalia, Artiodactyla) aus den Dinotheriensanden (Unterpliozän = Pont) Rheinhessens (Südwestdeutschland). Mémoires suisses de Paléontologie 86: 1–96.
9. Orliac M (2006) *Eurolistriodon tenarezensis*, sp. nov., from Montréal-du-Gers (France): implications for the systematics of the European Listriodontinae (Suidae, Mammalia). Journal of Vertebrate Paleontology 26: 967–980.
10. Orliac M (2007) Le rôle des Listriodontinae dans la différenciation des Suidae (Mammalia) ; paléoanatomie, systématique, phylogénie. Paris: Université Pierre et Marie Curie - Paris VI. pp. 702.
11. Smith JB,Dodson P (2003) A proposal for a standard terminology of anatomical notation and orientation in fossil vertebrate dentitions. Journal of Vertebrate Paleontology 23: 1–12.
