# Supplementary material for: A New Species of Nyanzachoerus (Cetartiodactyla: Suidae) from the Late Miocene Toros-Ménalla, Chad, Central Africa
Source: PLoS One. 2014 Aug 27;9(8):e103221. doi: 10.1371/journal.pone.0103221 (PMC4146473; doi:10.1371/journal.pone.0103221)
Supplement: Text S3 — Definition of complexity scores (CS). (PDF) [file pone.0103221.s012.pdf]

Text S3. Definition of complexity scores (CS).

The complexity scores (CS) for upper and lower third molars are defined in the list below and illustrated in Fig. 1S. Dental nomenclature follows [1].

Upper Third Molars

CS=0.75
Distocone reduced by comparison with trigon cusps; possible occurrence of small styles.

CS=1.0
Distocone strong, similar in size with trigon cusps; reduced postectostyles, not reaching distally beyond the first halft of the distocone; no well-developed postentostyles.

CS=1.2
Distocone as for 1.0; postectostyles more developed, reaching the distal half of the distocone; alternately, postectostyles as in 1.0 and postentostyle well-developed.

CS=1.4
All structures as 1.2, with in addition loss of contact between the metacone and the distocone, with intercalation of a postentostyle or a basin.

CS=1.6
Postectostyles reaching close to the distal extremity of the crown and distocone can be lingually shifted.

CS=1.8
Distocone dominated by a major development of postectostyles, covering a wider area than the distocone and reaching (even slightly) more distally.

CS=2.0
Distal postectostyle almost as large as the distocone and markedly stronger than other postectostyles, which are many.

CS=2.5
Two distocone of equivalent size or distal distocone stronger, with numerous postectostyles.

Lower Third Molars

CS=0.75
Distoconid reduced by comparison with hypo- and entoconid; possible occurrence of small poststylids.

CS=1.0
Distoconid strong, similar in size with hypo- and entoconid; poststylids tiny or missing.

CS=1.25

Distoconid as 1.0; poststylids more developed, but not reaching distally beyond the first half of the distoconid.

CS=1.5

Distoconid as for 1.0; poststylids more developed, reaching beyond the distal half of the distocone.

CS=1.75

Distoconid generally shifted; numerous and/or strong poststylids, reaching the distal extremity of the crown without passing the distoconid.

CS=2.0

Two distoconids subequal in size, most often organized in a symmetrical pair (hereafter named “a pair”); alternately, numerous and/or strong poststylids, passing the distal extremity of the distoconid but without forming together a clearly smaller area than that of the distoconid.

CS=2.25

Numerous and/or strong poststylids slightly passing the distal extremity of the distoconid, each poststylid much smaller than the distoconid, but forming together an area at list equivalent to that of the distoconid.

CS=2.5

A pair accompanied by a smaller stylid positioned more or less distally to the pair.

CS=2.75

A pair with strong and/or numerous stylids; alternaly a pair with a single large stylid positioned in the tooth main axis.

CS=3.0

A pair accompanied by a third distoconid centered and equivalent in size to the pair distoconids, without poststylids; alternately, a pair and a series of smaller stylids that have together an area comparable to that of the pair largest cuspid; alternately, a pair reduced compared to the hypo- and entoconid and a series of smaller stylids that have together an area larger than that of the pair largest cuspid.

CS=3.25

A pair accompanied by a third distoconid centered and equivalent in size to the pair distoconids with small poststylids.

CS=3.5

A pair accompanied by a third distoconid centered and equivalent in size to the pair distoconids with strong poststylids not reaching the distal extremity of the crown.

CS=3.75

A pair accompanied by a third distoconid equivalent in size to the pair distoconids, with strong poststylids reaching or passing the distal extremity of the crown; alternately a pair and a variable number of strong stylids that have together an area much larger than that of the pair largest cuspid.

CS=4.0
Two pairs.

CS=5.0
Two pairs accompanied by a fifth distoconid centered and equivalent in size to the pair distoconids.

References

1. Boisserie J-R, Lihoreau F, Orliac M, Fisher RE, Weston EM, et al. (2010) Morphology and phylogenetic relationships of the earliest known hippopotamids (Cetartiodactyla, Hippopotamidae, Kenyapotaminae). Zoological Journal of the Linnean Society 158: 325–366.
